# Estimation of COVID-19 Period Prevalence and the Undiagnosed Population in Canadian Provinces: Model-Based Analysis

**DOI:** 10.2196/26409

**Published:** 2021-09-09

**Authors:** Abdullah Hamadeh, Zeny Feng, Jessmyn Niergarth, William WL Wong

**Affiliations:** 1 School of Pharmacy University of Waterloo Kitchener, ON Canada; 2 Department of Mathematics and Statistics University of Guelph Guelph, ON Canada

**Keywords:** COVID-19, prevalence, undiagnosed proportion, mathematical modeling, estimate, Canada, diagnosis, control, distribution, infectious disease, model, framework, progression, transmission

## Abstract

**Background:**

The development of a successful COVID-19 control strategy requires a thorough understanding of the trends in geographic and demographic distributions of disease burden. In terms of the estimation of the population prevalence, this includes the crucial process of unravelling the number of patients who remain undiagnosed.

**Objective:**

This study estimates the period prevalence of COVID-19 between March 1, 2020, and November 30, 2020, and the proportion of the infected population that remained undiagnosed in the Canadian provinces of Quebec, Ontario, Alberta, and British Columbia.

**Methods:**

A model-based mathematical framework based on a disease progression and transmission model was developed to estimate the historical prevalence of COVID-19 using provincial-level statistics reporting seroprevalence, diagnoses, and deaths resulting from COVID-19. The framework was applied to three different age cohorts (< 30; 30-69; and ≥70 years) in each of the provinces studied.

**Results:**

The estimates of COVID-19 period prevalence between March 1, 2020, and November 30, 2020, were 4.73% (95% CI 4.42%-4.99%) for Quebec, 2.88% (95% CI 2.75%-3.02%) for Ontario, 3.27% (95% CI 2.72%-3.70%) for Alberta, and 2.95% (95% CI 2.77%-3.15%) for British Columbia. Among the cohorts considered in this study, the estimated total number of infections ranged from 2-fold the number of diagnoses (among Quebecers, aged ≥70 years: 26,476/53,549, 49.44%) to 6-fold the number of diagnoses (among British Columbians aged ≥70 years: 3108/18,147, 17.12%).

**Conclusions:**

Our estimates indicate that a high proportion of the population infected between March 1 and November 30, 2020, remained undiagnosed. Knowledge of COVID-19 period prevalence and the undiagnosed population can provide vital evidence that policy makers can consider when planning COVID-19 control interventions and vaccination programs.

## Introduction

The epidemiological information used to plan and evaluate strategies to prevent the spread of COVID-19 has undergone rapid changes since the start of the pandemic. As with many countries across the world, most Canadian provinces have enforced nonpharmaceutical interventions (NPIs) such as travel bans, school closures, and restrictions on nonessential businesses, workplaces, and social gatherings [1-4]. These measures, coupled with the highly uncertain and life-threatening nature of the disease, have resulted in profound societal and economic impacts [5].

With the high number of observed symptomless cases [3,4,6-12], there is now strong evidence that COVID-19 remains asymptomatic in a significant proportion of the infected population. This feature of the disease has been hypothesized to be a main driver of the rapid spread of COVID-19 worldwide [1,2]. Studies have also demonstrated that the transmissibility of SARS-CoV-2, the causative agent of COVID-19 via asymptomatic and symptomatic individuals is similar [13,14].

With the emergence of new, more transmissible variants of SARS-CoV-2 [15] and given the age-dependence of the likelihood of transmission and hospitalization following infection [16,17], the development of a successful COVID-19 control strategy requires a thorough understanding of the trends in the geographic and demographic distribution of disease burden. Estimation of the undiagnosed population is crucial for the planning and allocation of resources needed to implement restrictions for preventing disease spread and, more importantly, for knowing when it is appropriate to relax such restrictions. In addition, knowledge of the size of the previously infected population is of importance for estimating the remaining susceptible population and for planning next-generation vaccination drives in response to emerging variants [18].

In Canada, data on COVID-19 prevalence that include the undiagnosed population are extremely limited. Estimates of prevalence of the disease have included seroprevalence studies [19-24]. These likely underestimate the true COVID-19 prevalence owing to small sample sizes and an undersampling of the groups that are most affected by COVID-19, such as lower socioeconomic status groups and immigrant groups [25,26]. Alternatively, COVID-19 prevalence and incidence can be inferred using a back-calculation approach [27,28], in which recently observed occurrences of COVID-19–related late-stage events (eg, COVID-19–related deaths) are mapped backward using a mathematical simulation model of the natural history of the disease. An important advantage of the back-calculation approach over others is its ability to include the undiagnosed population in the prevalence estimation.

Our objective is to estimate the period prevalence of COVID-19 between March 1 and November 30, 2020, and the proportion of the infected population that remained undiagnosed in the Canadian provinces of Quebec (QC), Ontario (ON), Alberta (AB), and British Columbia (BC) by using a model-based back-calculation framework. These estimates are derived for three different age cohorts (under 30; 30-69; and ≥70 years) in each of the provinces studied. These provinces are the four most populated in Canada and were selected because the vast majority of COVID-19 cases in Canada were observed in these geographic regions across the study period.

This study presents a framework for estimating the disease burden by region, demographics, and diagnosis status. A disease progression and transmission model is used to estimate the size and composition of the COVID-19 period prevalence by integrating the results of previous seroprevalence surveys with primary provincial observed data of health events related to COVID-19 and its sequelae, including COVID-19–related deaths. From these estimates, we derive the proportion of the infected population that remained undiagnosed during this period.

## Methods

### Overview

A mathematical framework based on a compartmental disease progression and transmission model was developed for the estimation of the period prevalence for a given population. The framework was applied to COVID-19 data from each of QC, ON, AB, and BC. For each province, a Markov chain Monte Carlo (MCMC)–based Bayesian state estimation algorithm [29] was used to construct joint posterior probability distributions for the unknown model parameters and the daily number of individuals in each COVID-19 health state. These probability distributions are constructed by iteratively comparing the model-generated mean estimates of the daily numbers of COVID-19–related health events and period prevalence against observed calibration targets. The calibration targets were obtained from provincial data collected between March 1 and November 30, 2020, that reported (1) daily cases of newly diagnosed COVID-19 [30-33], (2) daily new deaths attributed to COVID-19, and (3) COVID-19 seroprevalence [21-24]. An overview of our proposed method is presented in the following subsections. A detailed methodology section is included in Multimedia Appendix 1.

### Disease Progression Model Assumptions

**Figure 1:**
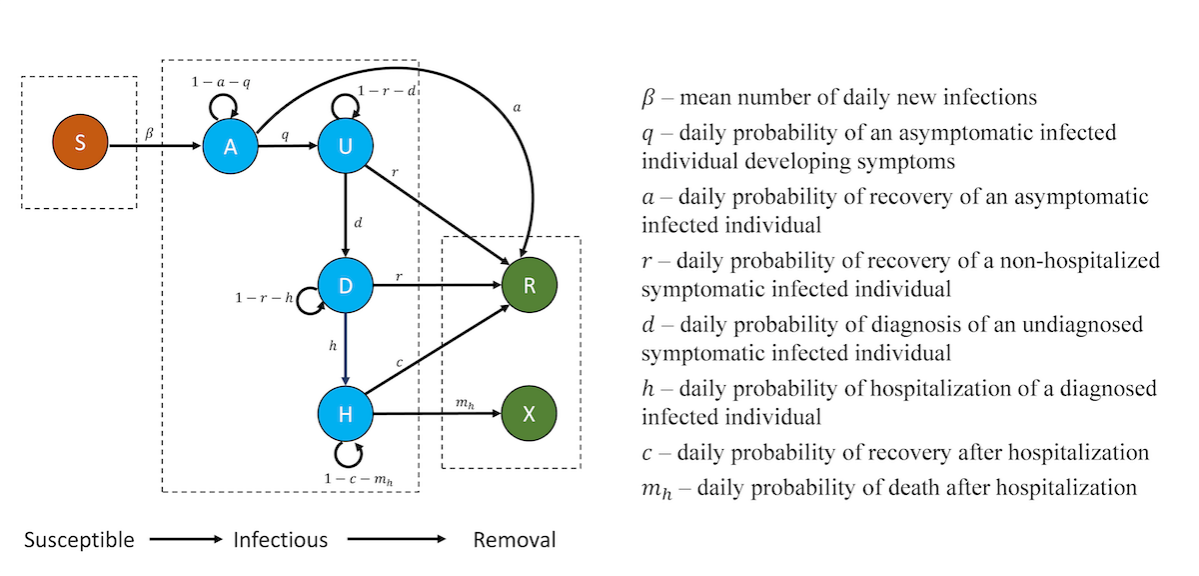
COVID-19 model conceptual diagram. Health states: Susceptible (S); asymptomatic and undiagnosed (A); symptomatic and undiagnosed (U); symptomatic and diagnosed (D); hospitalized (H); recovered (R); and death (X).

For each province, we develop an age-stratified “susceptible-infectious-removed” (SIR) compartmental framework to describe the progression through various disease states for individuals of the population. We stratified each population into three age cohorts: <30, 30 to 69, and ≥70 years. The model is structured based on the COVID-19 natural history model illustrated in Figure 1. The infectious state is subdivided into 4 health states: (1) state A, representing infected individuals who show no symptoms and are undiagnosed; (2) state U, representing symptomatic and undiagnosed individuals; (3) state D, representing individuals who are symptomatic and diagnosed; and (4) state H, representing hospitalized individuals. Individuals who recover (R) or die (X) are considered to be in the removal state.

We assume that individuals who reach state D do so by progressing through the states A → U → D. We also assume that the daily probability of recovery of an infected individual depends on their age cohort and whether they are in state A, state U or D, or state H. We assume all deaths due to COVID-19 are diagnosed and hospitalized prior to death. We assume that COVID-19–related mortality decreases gradually over time for all age groups as a result of better understanding of the disease and that the daily probability of diagnosing a COVID-19 infection has increased gradually since the start of the pandemic as a result of improved testing capacity.

### Disease Transmission Dynamics Assumptions

Within each province, we assume the disease to be transmissible across different age cohorts. We assume that the infectiveness of an infected individual will depend on whether they have been diagnosed (due to self-isolation following a diagnosis) and on whether they show symptoms [34]. Thus, the mean number of daily new infections caused by an infected individual will vary depending on their health state (A, U, or D).

Canadian Provinces have implemented NPIs to combat the spread of COVID-19. These interventions include travel bans, closure of schools and nonessential businesses, and limits on social gatherings. To reflect the effects of NPIs, seasonal effects on the transmissibility of SARS-CoV-2, and changes in public behavior, the infection rates *K_A_*, *K_U_*, and *K_D_* are allowed to vary over 9 different periods: The first three periods (March 1-11, March 12- 29, and March 30 to June 1, 2020) reflect the periods of preimplementation, partial implementation, and full implementation of NPIs, respectively. The latter periods correspond to each of the months of June to November 2020. The daily probability of diagnosis of infected individuals is assumed to have increased gradually between March 1 and November 30, 2020, reflecting increases in testing capacity across this period.

### Model Fitting

Health event data reporting the daily numbers of diagnosed cases and COVID-19–related deaths were collected for each province and age group for the study period. A summary of the cumulative diagnoses and COVID-19–related deaths as of November 30, 2020, is provided in Table 1.

Statistics on rates of recovery, testing, and hospitalization across the study period were also collected [30-33]. From these data, initial estimates of the mean values of the daily probabilities of hospitalization for diagnosed cases, as well as the daily probabilities of recovery and death for diagnosed and hospitalized cases, were calculated. Initial estimates of the mean daily probability of developing symptoms and of being discharged from the hospital were obtained from the literature [35,36]. The Metropolis-Hastings MCMC (MH-MCMC) algorithm was used to calibrate the remaining unknown parameters (Multimedia Appendix 2), and Kalman filtering was used to calibrate the daily number of individuals within each health state. The negative sum of the square of the weighted differences between (1) the expected and observed daily numbers of confirmed cases and deaths and (2) the expected and observed seroprevalence (Table 2) was used to approximate the log-likelihood function for computing the posterior distributions of unknown parameters and the unobserved daily numbers of individuals in each health state.

**Table 1:** Cumulative observed COVID-19 diagnoses and deaths as per data for Quebec, Ontario, Alberta, and British Columbia as of November 30, 2020.

Province and age cohort (years)		Population	Cumulative diagnoses as of November 30, 2020	Cumulative deaths as of November 30, 2020
**Quebec**				
	<30	2,829,745	45,778	4
	30-69	4,534,110	71,658	584
	≥70	1,121,110	26,476	6467
**Ontario**				
	<30	5,227,392	38,464	0
	30-69	7,607,840	62,331	471
	≥70	1,731,315	15,692	3084
**Alberta**				
	<30	1,674,906	23,207	3
	30-69	2,314,614	32,223	67
	≥70	381,796	4013	491
**British Columbia**				
	<30	1,681,252	12,054	0
	30-69	2,747,468	18,624	67
	≥70	642,616	3108	374

**Table 2:** Canadian provincial COVID-19 seroprevalence surveys conducted between March 2020 and July 2020 used for model fitting.

Province and survey date		Seroprevalence				Reference
		Age adjusted % (95% CI)	Total assays, n	Positive assays, n (%)		
**Quebec**						
	July 9, 2020	2.23 (1.90-2.56)	7691	173 (2.25)	[22]	
**Ontario**						
	April 30, 2020	0.5 (0.1-1.5)	827	3 (0.36)	[21]	
	May 31, 2020	1.5 (0.7-2.2)	1061	15 (1.41)	[21]	
	June 18, 2020	0.96 (0.810-1.113)	19,839	189 (0.95)	[23]	
	June 30, 2020	1.1 (0.8-1.3)	7014	79 (1.12)	[21]	
**Alberta**						
	June 18, 2020	0.37 (0.182-0.552)	5644	24 (0.42)	[23]	
**British Columbia**						
	March 13, 2020	0.28 (0.03-0.95)	869	2 (0.23)	[24]	
	May 27, 2020	0.55 (0.15-1.37)	885	4 (0.45)	[24]	
	June 18, 2020	0.50 (0.304-0.694)	4962	29 (0.58)	[23]	

### Model Validation

The disease progression model was used to back-calculate each cohort’s COVID-19 period prevalence based on the reported confirmed cases and deaths shown in Table 1, in addition to early provincial seroprevalence results as reported in Table 2. The fitted models generally showed close agreement with these data across the four provinces and three age cohorts. Multimedia Appendix 3 shows the fit of the models to the reported daily numbers of confirmed cases and deaths.

Table 3 summarizes the seroprevalence in Ontario for the months of July and August 2020 [19,20], which was reported by Public Health Ontario to be 1.1% (0.8%-1.3%). These two latter reported seroprevalences were not used as observed data in the model fitting but were instead used to validate the fitted model. Our calibrated model for Ontario showed close agreement with these latter seroprevalence survey results, with an estimated mean period prevalence of 1.13% as of July 31 (164,740 cases) and 1.20% (175,050 cases) as of August 31.

**Table 3:** Provincial COVID-19 seroprevalence surveys between July 2020 and August 2020 used for model validation in Ontario, Canada.

Date	Seroprevalence				Reference
	Age adjusted % (95% CI)	Total assays, n	Positive assays, n (%)		
July 31, 2020	1.1 (0.8-1.3)	7001	70 (0.99)	[20]	
August 31, 2020	1.1 (0.8-1.3)	6789	72 (1.06)	[19]	

## Results

### Prevalence and Total Incidence Estimates

**Figure 2:**
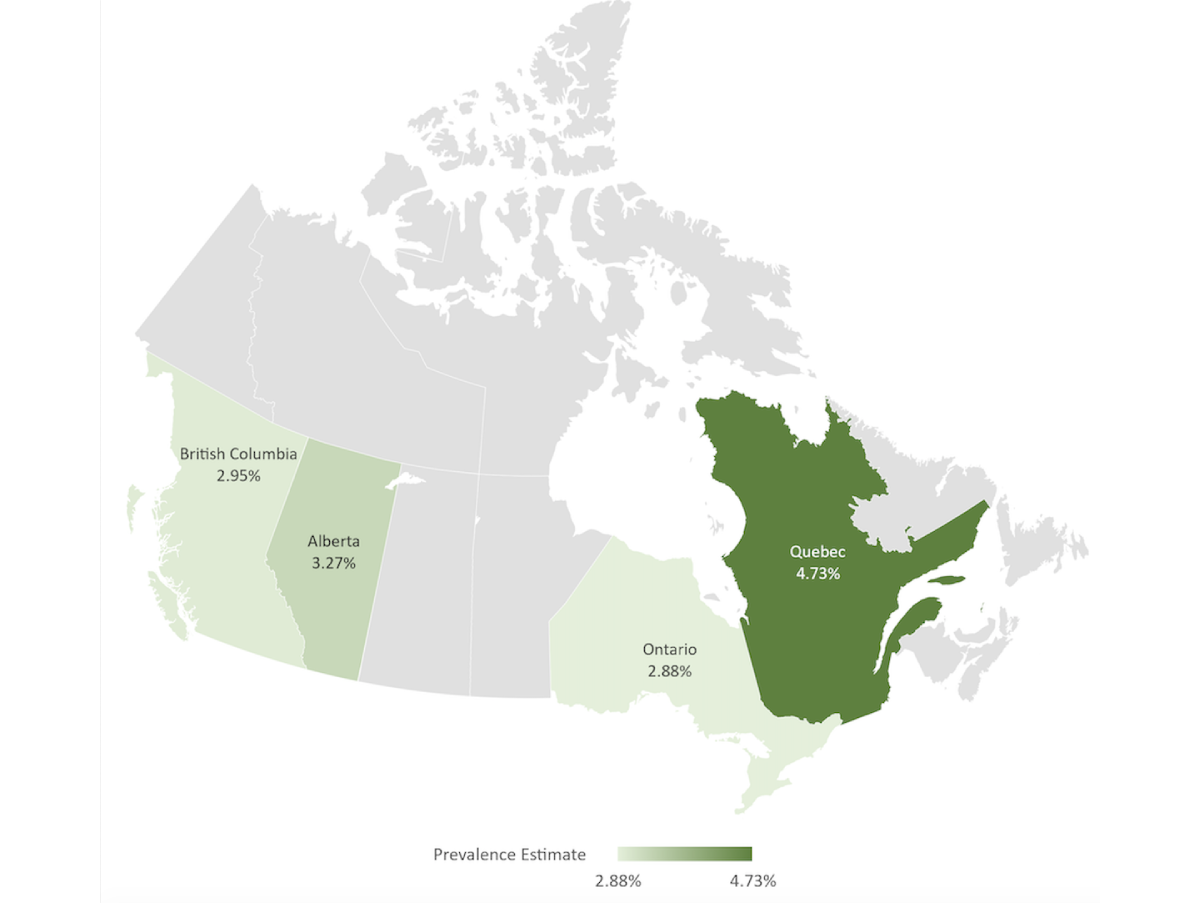
Estimated COVID-19 period prevalence in Quebec, Ontario, Alberta, and British Columbia between March 1 and November 30, 2020.

The mean estimates of the COVID-19 period prevalence between March 1 and November 30, 2020, for each province are as follows: 4.73% (95% CI 4.42%-4.99%) for QC, 2.88% (95% CI 2.75%-3.02%) for ON; 3.27% (95% CI 2.72%-3.70%) for AB; and 2.95% (95% CI 2.77%-3.15%) for BC, as illustrated in Figure 2.

**Figure 3:**
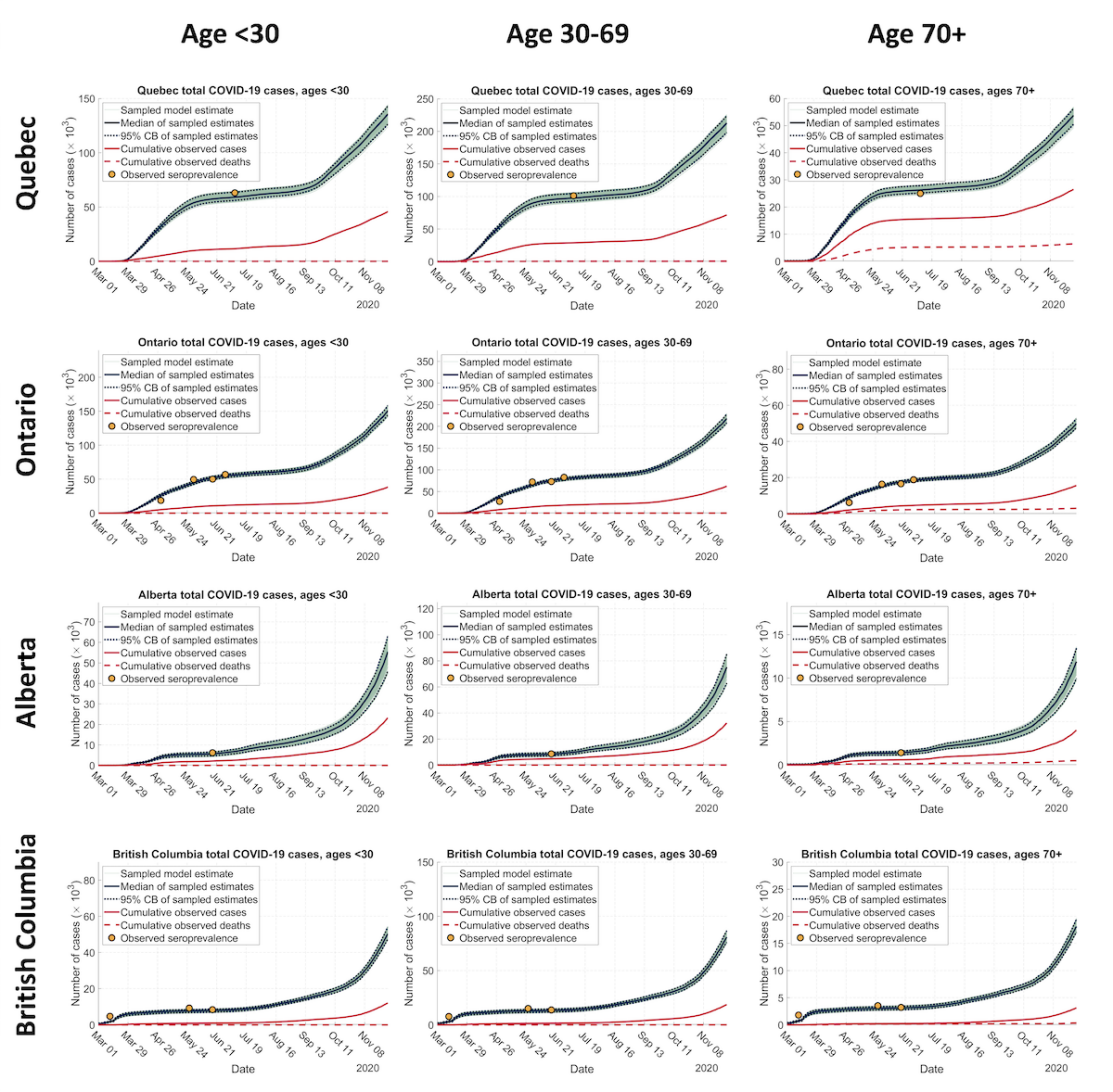
Estimated trajectories of cumulative total COVID-19 cases, cumulative reported COVID-19 diagnoses, and cumulative reported COVID-19 deaths in Quebec, Ontario, Alberta, and British Columbia between March 1 and November 30, 2020. CB: credible band.

Figure 3 shows the observed and estimated cumulative total number of infections for each age cohort up to November 30, 2020. For that date, the median estimates of the cumulative total numbers of infected individuals were as follows: 135,407 (95% CI 126,380-143,185) for QC, 151,443 (95% CI 144,707-158,804) for ON, 55,596 (95% CI 45,892-63,063) for AB, and 50,356 (95% CI 47,318-53,912) for BC, among individuals aged under 30 years; 212,048 (95% CI 198,212-223,863) for QC, 218,446 (95% CI 208,519-228,609) for ON, 75,246 (95% CI 62,932-85,136) for AB, 80,915 (95% CI 76,063-86,605) for BC, among individuals aged between 30 and 69 years; and 53,549 (95% CI 50,462-56,298) for QC, 49,937 (95% CI 47,614-52,440) for ON, 11,932 (95% CI 9,961-13,445) for AB, 18,147 (95% CI 17,076-19,397) for BC, among individuals aged 70 years or above. Multimedia Appendix 4 shows the estimated distributions of the cumulative total number of individuals with COVID-19 infection and the reported cumulative total number of individuals diagnosed with COVID-19 infection. Summary statistics for the distributions are tabulated in Multimedia Appendix 5. These estimates include both the diagnosed and undiagnosed populations and they range between 2 and 6 times the reported diagnoses of the provinces as, illustrated in Figure 3.

### Undiagnosed Proportion

Across the study period, the estimated proportion of the total infected population that was undiagnosed in each cohort and province was as follows: 66.19% (95% CI 63.78%-68.03%) for QC, 74.60% (95% CI 73.42%-75.78%) for ON, 58.26% (95% CI 49.43%-63.20%) for AB, and 76.06% (95% CI 74.53%-77.64%) for BC among individuals under 30 years; 66.21% (95% CI 63.85%-67.99%) for QC, 71.47% (95% CI 70.11%-72.73%) for ON, 57.18% (95% CI 48.80%-62.15%) for AB, and 76.98% (95% CI 75.52%-78.50%) for BC among individuals aged between 30 and 69 years; and 50.56% (95% CI 47.53%-52.97%) for QC, 68.58% (95% CI 67.04%-70.08%) for ON, 66.37% (95% CI 59.71%-70.15%) for AB, and 82.87% (95% CI 81.80%-83.98%) for BC among individuals aged 70 years and above.

## Discussion

### Principal Findings

We have combined our mathematical model with detailed province-level data to provide a comprehensive and robust estimate of the burden of COVID-19 infections in four large Canadian provinces between March 1 and November 30, 2020. Across all provinces and cohorts studied, our model-based prevalence estimates indicate that the period prevalence from March 1 to November 30, 2020, including both the diagnosed and undiagnosed populations, ranged between 2- to 6-fold the reported diagnosed period prevalence.

A variety of methods have previously been used to estimate the true prevalence of infectious diseases in Canada and around the world, including that of hepatitis C and HIV [37-41]. Common methods include seroprevalence surveys and model-based approaches [39]. Our findings suggest that the prevalence of COVID-19 in Canada over the study period was significantly higher than estimates based on data reported by provinces. In comparison, our estimates are congruent with seroprevalence surveys [3,4,6,42,43] and model-based approaches [27,28] conducted elsewhere around the world. Specifically, Bajema et al [42] reported the results of a large-scale seroprevalence survey conducted in the United States and found that the seroprevalence of New York state can be as high as 23%, much higher than the reported cases. Rostami et al [43] reported a systematic review and meta-analysis of seroprevalence of 23 countries worldwide and concluded that over 263 million people had been exposed or infected with COVID-19 as of the end of August 2020, roughly 10 times more than the 25 million people reported [44]. Flaxman et al [27] generated a model-based prevalence estimate based on a back-calculation method for 11 European countries and concluded that there are considerably fewer COVID-19 cases detected than their model estimated due to the presence of asymptomatic or mild cases. Perkins et al [28] also used a mathematical modeling approach to estimate the unobserved incidence in the United States and reported that the number of detected symptomatic infections was less than 10% of the total infected population during the early stage of the pandemic. As testing capacity has increased over time, the daily probability of diagnosis of a given infected individual will also have increased. Consequently, it is to be expected that the ratio between the overall prevalence and estimates of prevalence based solely on diagnosis figures will be lower than what has been reported for earlier stages of the pandemic. Our 2- to 6-fold estimates reasonably reflect this fact.

In contrast to the cross-sectional “snapshots” of the pandemic offered by seroprevalence surveys, our model-based approach provides longitudinal estimates of the COVID-19 population, which unveils the trends in the true spread of the disease over time as well as insights into the medium- to long-term effectiveness of NPIs in limiting transmissions. On the other hand, our analysis is subject to certain limitations. First, accurately estimating the period prevalence depends on knowledge of key model parameters such as the transmission rates and probabilities of diagnosis. The initial values of these parameters that were used in our model may have inherited biases from the existing literature on the natural history of COVID-19. However, through the Bayesian approach, uncertainties in these parameters are ultimately reflected in the credible intervals of the final prevalence estimates. Second, our method can, in principle, be applied to Canadian regions not considered in this study as well as to other countries. However, our longitudinal estimates of the period prevalence require high-resolution time-series data on the number of confirmed cases and deaths in each region and age cohort under investigation.

Establishing a robust baseline estimate of the prevalence and undiagnosed proportion is critical, as it contains important information for decision makers to plan for the future regarding how many individuals are likely to require vaccination and how much extra screening effort is needed to diagnose unaware infected individuals to prevent transmission. Our study provided estimates from the period of March 1 to November 30, 2020. Towards the end of the study period, COVID-19 vaccines were on track for deployment around the globe [45]. In recent months, these vaccines have been proven to be highly effective [46]. At the same time, new SARS-CoV-2 variants have reversed downward trends in infections even in countries with good rates of vaccination coverage [15], where these rapidly changing circumstances have prompted a re-evaluation of plans to ease NPIs. Given the higher transmissibility of the novel SARS-CoV-2 variants, updated estimates of the prevalence and undiagnosed proportion will be necessary, as this information contains important indicators for decision-makers to plan for future interventions, such as the distribution of next-generation vaccines [18].

If testing costs and time continue to decrease, expanding the number of tests can increase the diagnosis rate and reduce potential asymptomatic transmission [47]. However, to determine the appropriate level of expansion, such as, for example, whether to target specific high-risk populations or to conduct a general population screening, a cost-effectiveness analysis and budget impact analysis would be required [48]. Our framework for the inference of COVID-19 prevalence would provide pivotal parameters and estimates for these analyses.

### Conclusions

Our study provides a framework for estimating the prevalence of COVID-19 in Canada and indicates a substantial proportion of the population infected between March 1 and November 30, 2020, remained undiagnosed. The analysis we have presented provides a more complete picture of the pandemic than would be indicated from observations that only focus on COVID-19 diagnosis statistics. This information is critical for policy makers and public health officials when considering the implementation or relaxation of interventions for controlling COVID-19.

## Appendix

Multimedia Appendix 1 Detailed methodology section.

Multimedia Appendix 2 Model parameters and their Bayesian estimates.

Multimedia Appendix 3 Model validation results.

Multimedia Appendix 4 Distribution of the estimated cumulative total number of COVID-19 cases, reported cumulative diagnoses, and reported cumulative deaths.

Multimedia Appendix 5 Estimated cumulative total COVID-19 infections as of November 30, 2020.

## Abbreviations

AB: Alberta
BC: British Columbia
MCMC: Markov chain Monte Carlo
MH-MCMC: Metropolis-Hastings Markov chain Monte Carlo
NPIs: nonpharmaceutical interventions
ON: Ontario
QC: Quebec
SIR: susceptible-infectious-removed
