# Supplementary material for: Estimation of COVID-19 Period Prevalence and the Undiagnosed Population in Canadian Provinces: Model-Based Analysis
Source: JMIR Public Health Surveill. 2021 Sep 9;7(9):e26409. doi: 10.2196/26409 (PMC8432517; doi:10.2196/26409)
Supplement: Multimedia Appendix 1 [file publichealth_v7i9e26409_app1.docx]

**Supplementary Appendix 1:** Detailed Methodology Section

A mathematical framework based on a disease progression and transmission model was developed for the estimation of the daily COVID-19 prevalence in a given population. The framework was applied to QC, ON, AB and BC, which have been notably impacted by COVID-19. In our analysis, each province was considered to be a single population. For each population, we back-calculated the historical incidence of COVID-19 through a Markov chain Monte Carlo (MCMC)–based Bayesian state estimation approach. This approach was used to construct the joint posterior probability distributions for the unknown and uncertain model parameters, with the Metropolis-Hastings (M-H) algorithm used to sample parameters from these probability distributions. At each iteration of the M-H algorithm, given a set of proposed model parameters, Kalman filtering was used to estimate the daily mean numbers of health events related to COVID-19 infection [1]. The proposed calibration process constructs probability distributions of the historical incidence of COVID-19 by comparing the model-generated estimates of the daily mean numbers of health events and sequelae against observed calibration targets. These calibration targets were obtained from provincial data covering March 1, 2020 to November 30, 2020 that reported 1) daily new and total diagnosed COVID-19 cases, 2) daily new and total deaths attributed to COVID-19, 3) COVID-19 seroprevalence rates. Details of our proposed method will be presented in the following subsections.

**S1.1 | Disease progression model assumptions**

For a given population, we develop an age-stratified “susceptible-infectious-removal” (SIR) compartmental framework to describe the progression of the disease states for individuals within the population. We stratify each population into three age cohorts: <30, 30–69, and ≥ 70. The infected population is assumed to be distributed over an additional set of pre-defined health states. The size of the population within each health state evolves in daily time steps as infected individuals progress through different health states. The model is developed based on the natural history of the disease model as illustrated in FIGURE 1. The population size in each disease state is tracked for each age group at every time point *t* and we adopt a notation such that, for example, *S_i_(t)* represents the population size of the *i*^th^ age group (for *i* = 1, 2, 3) in the susceptible state *S* (in FIGURE 1) at time *t*. However, for simplicity, we suppress these notations (e.g. *S_i_(t)* is expressed as *S* in FIGURE 1).

In order for the model to incorporate age-dependent risks of developing symptoms the infectious state is sub-divided into four health states: asymptomatic and undiagnosed (A), symptomatic and undiagnosed (U), symptomatic and diagnosed (D), and hospitalized (H). Infected individuals showing no symptoms would be most likely in state A. Infected individuals showing mild symptoms/illness would be most likely in states U or D. Infected individuals showing severe/critical symptoms/illness would be most likely in states D or H. As the focus of our study is on the total prevalence of COVID-19, we do not consider the transition from hospital (non-intensive care unit) to the intensive care unit. Individuals who recover (R) or die (X) are considered to be in the removal state. We make the following assumptions:

- Most individuals showing COVID-19 symptoms are considered for the diagnostic test, so an infected diagnosed individual (D) would typically progress through the A → U → D states. The daily probability of diagnosis of infected individuals is assumed to have increased gradually between March 1 and November 30 to reflect the increase in testing rates. We denote this probability on those respective dates by *d_0_* and *d_1_*, where *d_0_* = *εd_1_* and 0<*ε* <1.
- We denote the daily probability of an infected asymptomatic individual (state A) developing symptoms by $q$. We assume that this probability increases with age. For an individual aged 70+, $q=\bar{q}$, where$0\leq\bar{q}\leq1.$ For an individual age 30-69, $q=\psi_{1}\bar{q}$, where$0\leq\psi_{1}\leq1.$For an individual aged below 30 years, $q=\psi_{2}\psi_{1}\bar{q}$, where$0\leq\psi_{2}\leq1.$
- We denote the probability of recovery without progression to a symptomatic state by $p_{a}$. We assume that this probability falls with age. For an individual aged below 30, $p_{a}=\bar{p}_{a}$, where$0\leq\bar{p}_{a}\leq1.$ For an individual age 30-69, $p_{a}={\gamma_{1}\bar{p}}_{a}$, where$0\leq\gamma_{1}\leq1.$For an individual aged 70+, $p_{a}={\gamma_{2}\gamma_{1}\bar{p}}_{a}$, where$0\leq\gamma_{2}\leq1.$Since an asymptomatic infected individual may only transition to the recovered state R or the symptomatic static U (with daily probability *q*) , the daily probability of recovery while asymptomatic, denoted $a$, is related to the cumulative probability of asymptomatic recovery, $p_{a}$, via the relation $p_{a}=a/(a+q)$.
- We denote the daily probability of recovery of a symptomatic individual (state U or D) by $r$. We assume that this probability falls with age. For an individual aged below 30, $r=\bar{r}$, where$0\leq\bar{r}\leq1.$ For an individual age 30-69, $r=\rho_{1}\bar{r}$, where$0\leq\rho_{1}\leq1.$For an individual aged 70+, $r=\rho_{2}\rho_{1}\bar{r}$, where$0\leq\rho_{2}\leq1.$
- We denote the daily probability of recovery of a hospitalized individual (in state H) by $c$. We assume that this probability falls with age. For an individual aged below 30, $c=\bar{c}$, where$0\leq\bar{c}\leq1.$ For an individual age 30-69, $c=\phi_{1}\bar{c}$, where$0\leq\phi_{1}\leq1.$For an individual aged 70+, $c=\phi_{2}\phi_{1}\bar{c}$, where$0\leq\phi_{2}\leq1.$
- We denote the daily probability of hospitalization of a diagnosed individual by $h$. We assume that this probability increases with age.
- We assume all deaths from the COVID-19 infection are diagnosed and hospitalized prior to death. We denote the daily probability of death of a hospitalized individual (in state H) on day *t* by $m_{h}(t)$. To account for improvements in treatment, we assume that $m_{h}(t)$ decreases piecewise linearly over eight different periods between March 1 and November 30, 2020. The daily probability $m_{h}(t)$ is thus given by

| *m_h_*(*t*)*=* |  | *m_h0_* | *t ≤ T_m0_* |
| --- | --- | --- | --- |
|  |  | *m_hi_ +* (*m_h_*_(_*_i+1_*_)_*-m_hi_*)(*t – T_mi_*)*/*(*T_m(i+1)_ – T_mi_*) | *T­_mi_ < t ≤ T­_m(i+1)_* for *i=0,…,5* |
|  |  | *m_h6_* | *t > T­_m6_* |

where $m_{h0}$ is the baseline daily probability of death of a patient in state H up to March 30 and

$m_{hj}=m_{h0}\prod_{i=1}^{j} \eta_{i}$, with$\eta_{i}\leq1$ for $j=1,\cdots,6$. The transition dates *T­_mi_* of this piecewise linear function correspond to: *T­_m0_*=March 30, *T­_m1_*=June 1, *T­_m2_*=July 1, *T­_m3_*=August 1, *T­_m4_*=September 1, *T­_m5_*=October 1, *T_m_­_6_*=November 1.

- At every time point, the susceptible population is taken to be the difference between the total population and the sum of the individuals in infectious and removal states.

**S1.2 | Disease transmission dynamics assumptions**

Within each province, we allow disease to be transmitted across different age cohorts. Once an infected individual is diagnosed (D), they will be requested to self-isolate, causing their infectivity to fall below that of undiagnosed infected individuals (in states A or U). Thus, the mean number of daily new infections caused by an infected individual will vary depending on the health state of the infected person. The mean number of new infections by individuals in state *j* (*j*=A, U, D) is assumed to be proportional to the number of individuals in state *j* and the proportion of the population that is susceptible, with the constants of proportionality denoted *K­_A_*, *K­_U_*, and *K_D_* respectively. The mean number of daily new infections on day $t$ among individuals in cohort *i* in a given province is estimated by $\beta$ (see FIGURE 1), where

$$\beta\left( t \right)=\frac{S_{i}\left( t \right)}{P}\left( K_{A}\left( t \right)A\left( t \right)+K_{U}\left( t \right)U\left( t \right)+K_{D}\left( t \right)D\left( t \right) \right),$$

$P$ is the provincial population, $S_{i}\left( t \right)$ is the cohort’s susceptible population, and $A$, $U$ and $D$ represent the number of individuals in the province in states A, U and D, respectively.

Due to the absence of a vaccine and effective treatments for COVID-19 during the study period, Canadian Provinces implemented NPIs to combat the disease’s spread that included travel bans, closures of schools and non-essential businesses, social distancing, reduced capacities in indoor public spaces, and recommendations to wear masks. To reflect the effects of NPIs on infection rates, *K­_A_*, *K­_U_*, and *K_D_* are assumed to vary piecewise linearly over nine different periods: The first three periods (March 1 to March 11, March 12 to March 29, and March 30 to June 1) reflect the periods before the implementation of NPIs, the gradual implementation of NPIs and the full implementation of NPIs respectively. The latter six periods correspond to the months of June to November, 2020. Letting *K_A_(t), K_U_(t), K_D_(t)* be the mean numbers of newly infected susceptible individuals being infected by infectious individuals in the A, U, and D health states, respectively, on day *t*, we have:

| *K_A_(t)=* |  | *K_A0_* | *t < T_0_* |
| --- | --- | --- | --- |
|  |  | *K_Ai_ + (K_A(i+1)_-K_Ai_)(t – T_i_)/(T_i+1_ – T_i_)* | *T­_i_ ≤ t < T­_i+1_* for *i=0,…,6* |
|  |  | *K_A7_* | *t ≥ T­_7_* |

| *K_U_(t)=* |  | *K_U0_* | *t < T_0_* |
| --- | --- | --- | --- |
|  |  | *K_Ui_ + (K_U(i+1)_-K_Ui_)(t – T_i_)/(T_i+1_ – T_i_)* | *T­_i_ ≤ t < T­_i+1_* for *i=0,…,6* |
|  |  | *K_U7_* | *t ≥ T­_7_* |

| *K_D_(t)=* |  | *K_D0_* | *t < T_0_* |
| --- | --- | --- | --- |
|  |  | *K_Di_ + (K_D(i+1)_-K_Di_)(t – T_i_)/(T_i+1_ – T_i_)* | *T­_i_ ≤ t < T­_i+1_* for *i=0,…,6* |
|  |  | *K_D7_* | *t ≥ T­_7_* |

Here, *K­_A0_*, *K­_U0_*, and *K_D0_* are the baseline values of the rates *K­_A_*, *K­_U_*, and *K_D_* respectively in the first of the nine periods (i.e. prior to March 12). The rates *K_Ai,_ K_Ui,_* and *K_Di_* for *i*= 1,…,7 are the values of *K­_A_*, *K­_U_*, and *K_D_* on transition days *T_i_*, where *T_0_ =* March 12, 2020*, T_1_ =* March 30, 2020 and *T_2_ =* June 1, 2020*, T_3_ =* July 1, 2020, *T_4_ =* August 1, 2020, *T_5_ =* September 1, 2020, *T_6_ =* October 1, 2020 and *T_7_ =* November 1, 2020 *.* The values of *K­_Ai_* and *K­_Ui_* are obtained by scaling *K­_A0_*, *K­_U0_* by constants that represent the effect of the imposition or relaxation of NPIs. As such, we assume that *K_Ai_= α _i_K_A0_, K_Ui_= σ _i_K_U0_* for *i*=1,…,7 where *α_i_*>0*, σ_i_*>0. Since the infectivity rates *K­_A_*, *K­_U_* are not directly measurable, we let the scale factors *α_i_, σ_i_* range between 0 and 8. Under this assumption, the infectivity rates *K_Ai_* and *K_Ui_* after the first transition (on March 11) can be up to eight-fold the infectivity seen at the start of the pandemic, when no NPIs were in place. In addition, diagnosed symptomatic patients are requested to quarantine, and we therefore assume their infectivity to be lower than that of undiagnosed individuals. We therefore let *K_Di_= δ_i_K_Ui_* where 0*≤δ_i_* ≤1*.*

As there is little evidence to suggest a gender-dependent effect on the transmissibility, infectivity, and the severity of the disease, we do not consider a gender effect in our model [2]. Transmission of the disease is assumed to only occur between individuals within the same geographic region, i.e., within a province. However, a truncated positive normally distributed random error term is included to capture random behavior within the provincial population that cannot be explained by the model, such as infections that occur due to infectious individuals from outside a given province infecting susceptible individuals within that province.

**S1.3 | Model Fitting**

Daily health event data reporting the daily numbers of newly diagnosed confirmed cases and COVID-19 related deaths were collected for the period from March 1 to November 30, 2020 for each of three age cohorts and each of the four provinces [3-6]. These statistics are summarized in TABLE 1. Provincial seroprevalence survey results dating between 13 March and 9 July, 2020 [7-10] were also collected and are summarized in TABLE 2.

Rates of recovery, testing, and hospitalization over the study period were also collected from the literature [11-14]. From these rates, initial estimates of the mean values of the daily probabilities of hospitalization for diagnosed cases, and the daily probabilities of recovery and death for diagnosed and hospitalized cases were calculated. Initial estimates of the mean daily probability of developing symptoms and of being discharged from hospital were obtained from the literature [15, 16]. The remaining unknown model parameters were: the province-specific parameters *K­_j0_*, for *j*= A, U, and D, scale factors *α_i_, σ_i_* and  *δ _i_* (*i*=1,…,7) in addition to the province and age-specific daily transition probabilities *q, p_a_ , r, h, c, m_h,_ d_0_* and *d_1_*, and the initial asymptomatic infected population size on March 1, denoted *A­(0).* The Metropolis-Hastings MCMC (MH-MCMC) algorithm was used to repeatedly sample these unknown parameters. At each iteration of the algorithm, new values of these parameters are proposed and Kalman filtering is used to estimate, conditional on the proposed parameters, each age group’s daily population in each health state *S(t), A­(t), U(t), D(t), H(t), R(t)* as well as the expected daily number of diagnosed cases and deaths from each age group. The acceptance rule of the proposed parameters in the MH algorithm is then constructed based on their posterior probability given the observed daily number of diagnoses and deaths in TABLE 1 and the reported seroprevalence results in TABLE 2 . The log-likelihood function used to compute the posterior probabilities was taken to be the negative sum of the square of the differences between the observed and the expected diagnoses, deaths and seroprevalence. This algorithm was implemented in MATLAB.

**References**

1. Zarchan P, Musoff H. Fundamentals of Kalman filtering : a practical approach. Reston, Va.: American Institute of Aeronautics and Astronautics; 2000. xx, 664 p. p. ISBN: 1563474557.

2. Bertocchi G. COVID-19 susceptibility, women, and work. VoxEU.org; 2020 [May 20, 2020]; Available from: <https://voxeu.org/article/covid-19-susceptibility-women-and-work>.

3. Government of Ontario. COVID-19 case data. Toronto, Ontario; 2020 [July 2, 2020]; Available from: <https://covid-19.ontario.ca/data>.

4. Gouvernement du Québec. Situation of the coronavirus (COVID-19) in Québec. Québec CIty; 2020 [July 2, 2020]; Available from: <https://www.quebec.ca/en/health/health-issues/a-z/2019-coronavirus/situation-coronavirus-in-quebec/>.

5. BC Centre for Disease Control. BC COVID-19 Data. Victoria, BC; 2020 [July 2, 2020]; Available from: <http://bccdc.ca/covid19data>.

6. Governement of Alberta. COVID-19 Alberta Statistics. Edmonton, AB; 2020 [July 2, 2020]; Available from: <https://covid19stats.alberta.ca>.

7. Héma-Québec. ÉTUDE DE SÉROPRÉVALENCE DES ANTI-SRAS-COV-2 CHEZ LES DONNEURS DE SANGD’HÉMA-QUÉBEC, VERS LA FIN DE LA PREMIÈRE VAGUE DECOVID-19–ÉTUDE NO. ET-20-004, PROJET COVID-20-02. Montreal, QC; 2020 [Nov 1, 2020]; Available from: <https://www.hema-quebec.qc.ca/userfiles/file/coronavirus/COVID-20-02-etude-seroprevalence-rapport-final.pdf>.

8. Public Health Ontario. COVID-19 Seroprevalence in Ontario: March 27, 2020 to June 30, 2020. Toronto, ON; 2020 [Nov 1, 2020]; Available from: <https://www.publichealthontario.ca/-/media/documents/ncov/epi/2020/07/covid-19-epi-seroprevalence-in-ontario.pdf?la=en>.

9. Canadian Blood Services. COVID-19 Seroprevalence Report – August 19, 2020. Ottawa, ON; 2020 [Nov 1, 2020]; Available from: <https://www.blood.ca/sites/default/files/CBS_COVID-19_Seroprevalence_Public_Report_Aug272020.pdf>.

10. Skowronski DM, Sekirov I, Sabaiduc S, Zou M, Morshed M, Lawrence D, et al. Low SARS-CoV-2 sero-prevalence based on anonymized residual sero-survey before and after first wave measures in British Columbia, Canada, March-May 2020. medRxiv. 2020 July 15.

11. Peeri NC, Shrestha N, Rahman MS, Zaki R, Tan Z, Bibi S, et al. The SARS, MERS and novel coronavirus (COVID-19) epidemics, the newest and biggest global health threats: what lessons have we learned? Int J Epidemiol. 2020 Feb 22. PMID: 32086938. doi: 10.1093/ije/dyaa033.

12. Zhao H, Lu X, Deng Y, Tang Y, Lu J. COVID-19: asymptomatic carrier transmission is an underestimated problem. Epidemiol Infect. 2020 Jun 11;148:e116. PMID: 32525469. doi: 10.1017/S0950268820001235.

13. Sood N, Simon P, Ebner P, Eichner D, Reynolds J, Bendavid E, et al. Seroprevalence of SARS-CoV-2-Specific Antibodies Among Adults in Los Angeles County, California, on April 10-11, 2020. JAMA. 2020 May 18. PMID: 32421144. doi: 10.1001/jama.2020.8279.

14. Goodman D, Rothfeld M. 1 in 5 New Yorkers May Have Had Covid-19, Antibody Tests Suggest. New York: New York Times; 2020 [May 20, 2020]; Available from: <https://www.nytimes.com/2020/04/23/nyregion/coronavirus-antibodies-test-ny.html>.

15. Lauer SA, Grantz KH, Bi Q, Jones FK, Zheng Q, Meredith HR, et al. The Incubation Period of Coronavirus Disease 2019 (COVID-19) From Publicly Reported Confirmed Cases: Estimation and Application. Ann Intern Med. 2020 May 5;172(9):577-82. PMID: 32150748. doi: 10.7326/M20-0504.

16. Li LQ, Huang T, Wang YQ, Wang ZP, Liang Y, Huang TB, et al. COVID-19 patients' clinical characteristics, discharge rate, and fatality rate of meta-analysis. J Med Virol. 2020 Jun;92(6):577-83. PMID: 32162702. doi: 10.1002/jmv.25757.
