# Supplementary material for: Estimation of COVID-19 Period Prevalence and the Undiagnosed Population in Canadian Provinces: Model-Based Analysis
Source: JMIR Public Health Surveill. 2021 Sep 9;7(9):e26409. doi: 10.2196/26409 (PMC8432517; doi:10.2196/26409)
Supplement: Multimedia Appendix 2 [file publichealth_v7i9e26409_app2.docx]

**Appendix 2:** Model parameters and their Bayesian estimates

| **Quebec** | | | |
| --- | --- | --- | --- |
| *Parameter* | | *Posterior* | *Prior* |
| Mean number of new infections per day by symptomatic and undiagnosed infected individuals per susceptible population fraction: *K_A0_, K_A1_,…, K_A7_*, where *K_Ai_= α _i_K_A0_*_,_ for *i=1,…,7.* | | | |
| *K_A0_* | | Mean: 0.07, 95% CI: 0.03-0.11 | Uniform(0.00,5.00) |
| *α_1_* | | Mean: 1.21, 95% CI: 0.89-1.38 | Uniform(0.00,8.00) |
| *α_2_* | | Mean: 0.79, 95% CI: 0.61-0.94 | Uniform(0.00,8.00) |
| *α_3_* | | Mean: 2.69, 95% CI: 2.51-2.80 | Uniform(0.00,8.00) |
| *α_4_* | | Mean: 3.14, 95% CI: 2.97-3.31 | Uniform(0.00,8.00) |
| *α_5_* | | Mean: 4.64, 95% CI: 4.49-4.94 | Uniform(0.00,8.00) |
| *α_6_* | | Mean: 3.11, 95% CI: 2.81-3.34 | Uniform(0.00,8.00) |
| *α_7_* | | Mean: 3.17, 95% CI: 2.98-3.32 | Uniform(0.00,8.00) |
| Mean number of new infections per day by symptomatic and undiagnosed infected individuals per susceptible population fraction: *K_U0_, K_U1_,…, K_U7_* , where *K_Ui_= σ _i_K_U0_*_,_ for *i=1,…,7.* | | | |
| *K_U0_* | | Mean: 0.16, 95% CI: 0.14-0.18 | Uniform(0.00,5.00) |
| *σ_1_* | | Mean: 3.22, 95% CI: 3.09-3.39 | Uniform(0.00,8.00) |
| *σ_2_* | | Mean: 1.92, 95% CI: 1.43-2.18 | Uniform(0.00,8.00) |
| *σ_3_* | | Mean: 1.63, 95% CI: 1.53-1.75 | Uniform(0.00,8.00) |
| *σ_4_* | | Mean: 1.83, 95% CI: 1.55-2.22 | Uniform(0.00,8.00) |
| *σ_5_* | | Mean: 2.56, 95% CI: 2.46-2.67 | Uniform(0.00,8.00) |
| *σ_6_* | | Mean: 3.36, 95% CI: 3.27-3.49 | Uniform(0.00,8.00) |
| *σ_7_* | | Mean: 3.02, 95% CI: 2.94-3.14 | Uniform(0.00,8.00) |
| Mean number of new infections per day by symptomatic and diagnosed infected individuals per susceptible population fraction: *K_D0_, K_D1_,…, K_D7_*, where *K_Di_= δ_i_K_Ui_*_,_ for *i=0,…,7.* | | | |
| *δ_0_* | | Mean: 0.62, 95% CI: 0.59-0.64 | Uniform(0.00,1.00) |
| *δ_1_* | | Mean: 0.08, 95% CI: 0.06-0.10 | Uniform(0.00,1.00) |
| *δ_2_* | | Mean: 0.41, 95% CI: 0.39-0.44 | Uniform(0.00,1.00) |
| *δ_3_* | | Mean: 0.38, 95% CI: 0.37-0.41 | Uniform(0.00,1.00) |
| *δ_4_* | | Mean: 0.32, 95% CI: 0.31-0.33 | Uniform(0.00,1.00) |
| *δ_5_* | | Mean: 0.35, 95% CI: 0.33-0.37 | Uniform(0.00,1.00) |
| *δ_6_* | | Mean: 0.18, 95% CI: 0.15-0.20 | Uniform(0.00,1.00) |
| *δ_7_* | | Mean: 0.33, 95% CI: 0.31-0.35 | Uniform(0.00,1.00) |
| Probability of recovery of an asymptomatic infected individual, *p_a_*.  Age < 30: *p_a_=* $\bar{p}_{a}$*.*  Age 30-69: *p_a_=* $\gamma_{1}\bar{p}_{a}$*.*  Age 70+: *p_a_=* ${{\gamma_{2}\gamma}_{1}\bar{p}}_{a}$*.* | | | |
| $\bar{p}_{a}$ | | Mean: 0.56, 95% CI: 0.55-0.57 | Uniform(0.50,0.88) |
| $\gamma_{1}$ | | Mean: 0.79, 95% CI: 0.79-0.80 | Uniform(0.50,0.80) |
| $\gamma_{2}$ | | Mean: 0.77, 95% CI: 0.76-0.77 | Uniform(0.50,0.80) |
| Daily probability of recovery of a symptomatic, non-hospitalized individual, *r.*  Age < 30: *r=* $\bar{r}$*.*  Age 30-69: *r=* $\rho_{1}\bar{r}$*.*  Age 70+: *r=* ${\rho_{2}\rho}_{1}\bar{r}$*.* | | | |
| $\bar{r}$ | | Mean: 0.18, 95% CI: 0.16-0.20 | Uniform(0.00,1.00) |
| $\rho_{1}$ | | Mean: 0.97, 95% CI: 0.94-0.99 | Uniform(0.00,1.00) |
| $\rho_{2}$ | | Mean: 0.30, 95% CI: 0.29-0.32 | Uniform(0.00,1.00) |
| Daily probability of recovery of a hospitalized individual, *c.*  Age < 30: *c=* $\bar{c}$*.*  Age 30-69: *c=* $\phi_{1}\bar{c}$*.*  Age 70+: *c=* ${\phi_{2}\phi}_{1}\bar{c}$*.* | | | |
| $\bar{c}$ | | Mean: 0.53, 95% CI: 0.51-0.54 | Uniform(0.00,1.00) |
| $\phi_{1}$ | | Mean: 0.53, 95% CI: 0.51-0.56 | Uniform(0.00,1.00) |
| $\phi_{2}$ | | Mean: 0.31, 95% CI: 0.29-0.34 | Uniform(0.00,1.00) |
| Daily probability of an asymptomatic infected individual developing symptoms, *q.*  Age < 30: *q=*${\psi_{2}\psi}_{1}\bar{q}$*.*  Age 30-69: *q=* $\psi_{1}\bar{q}$*.*  Age 70+: *q=* $\bar{q}$*.* | | | |
| $\bar{q}$ | | Mean: 0.80, 95% CI: 0.78-0.82 | Uniform(0.00,1.00) |
| $\psi_{1}$ | | Mean: 0.83, 95% CI: 0.81-0.84 | Uniform(0.00,1.00) |
| $\psi_{2}$ | | Mean: 0.64, 95% CI: 0.61-0.66 | Uniform(0.00,1.00) |
| Daily probability of diagnosis of an undiagnosed symptomatic individual in Age 70+ cohort: *d_0_* and *d_1_*, with *d*_0_= *εd_1_* | | | |
| *d_1_* | | Mean: 0.44, 95% CI: 0.43-0.44 | Uniform(0.00,0.50) |
| *ε* | | Mean: 0.16, 95% CI: 0.14-0.18 | Uniform(0.00,1.00) |
| Daily probability of hospitalization of a diagnosed symptomatic individual, *h* | | | |
| Age <30 | | Mean: 0.01, 95% CI: 0.01-0.01 | Uniform(0.00,0.05) |
| Age 30-69 | | Mean: 0.02, 95% CI: 0.00-0.05 | Uniform(0.00,0.05) |
| Age 70+ | | Mean: 0.04, 95% CI: 0.03-0.05 | Uniform(0.00,0.05) |
| Daily probability of death of a diagnosed, hospitalized individual, *m_h0_ …m_h6_, with* $m_{hj}=m_{h0}\prod_{i=1}^{j} \eta_{i}$*.* | | | |
| *m_h0_* | Age <30 | Mean: 0.00, 95% CI: 0.00-0.00 | Uniform(0.00,0.00) |
|  | Age 30-69 | Mean: 0.07, 95% CI: 0.01-0.18 | Uniform(0.00,0.20) |
|  | Age 70+ | Mean: 0.15, 95% CI: 0.10-0.20 | Uniform(0.00,0.20) |
| *η_1_* | *Age <30* | Mean: 0.70, 95% CI: 0.70-0.70 | Uniform(0.40,1.00) |
|  | *Age 30-69* | Mean: 0.72, 95% CI: 0.44-0.98 | Uniform(0.40,1.00) |
|  | *Age 70+* | Mean: 0.77, 95% CI: 0.54-0.97 | Uniform(0.40,1.00) |
| *η_2_* | *Age <30* | Mean: 0.70, 95% CI: 0.70-0.70 | Uniform(0.40,1.00) |
|  | *Age 30-69* | Mean: 0.65, 95% CI: 0.43-0.91 | Uniform(0.40,1.00) |
|  | *Age 70+* | Mean: 0.68, 95% CI: 0.42-0.94 | Uniform(0.40,1.00) |
| *η_3_* | *Age <30* | Mean: 0.70, 95% CI: 0.70-0.70 | Uniform(0.40,1.00) |
|  | *Age 30-69* | Mean: 0.69, 95% CI: 0.41-0.97 | Uniform(0.40,1.00) |
|  | *Age 70+* | Mean: 0.70, 95% CI: 0.43-0.94 | Uniform(0.40,1.00) |
| *η_4_* | *Age <30* | Mean: 0.70, 95% CI: 0.70-0.70 | Uniform(0.40,1.00) |
|  | *Age 30-69* | Mean: 0.73, 95% CI: 0.42-0.98 | Uniform(0.40,1.00) |
|  | *Age 70+* | Mean: 0.76, 95% CI: 0.52-0.96 | Uniform(0.40,1.00) |
| *η_5_* | *Age <30* | Mean: 0.70, 95% CI: 0.70-0.70 | Uniform(0.40,1.00) |
|  | *Age 30-69* | Mean: 0.66, 95% CI: 0.42-0.92 | Uniform(0.40,1.00) |
|  | *Age 70+* | Mean: 0.69, 95% CI: 0.44-0.96 | Uniform(0.40,1.00) |
| *η_6_* | *Age <30* | Mean: 0.70, 95% CI: 0.70-0.70 | Uniform(0.40,1.00) |
|  | *Age 30-69* | Mean: 0.72, 95% CI: 0.44-0.97 | Uniform(0.40,1.00) |
|  | *Age 70+* | Mean: 0.77, 95% CI: 0.45-0.98 | Uniform(0.40,1.00) |

| **Ontario** | | | |
| --- | --- | --- | --- |
| *Parameter* | | *Posterior* | *Prior* |
| Mean number of new infections per day by symptomatic and undiagnosed infected individuals per susceptible population fraction: *K_A0_, K_A1_,…, K_A7_*, where *K_Ai_= α _i_K_A0_*_,_ for *i=1,…,7.* | | | |
| *K_A0_* | | Mean: 0.29, 95% CI: 0.26-0.32 | Uniform(0.00,5.00) |
| *α_1_* | | Mean: 0.27, 95% CI: 0.12-0.44 | Uniform(0.00,8.00) |
| *α_2_* | | Mean: 1.49, 95% CI: 1.32-1.66 | Uniform(0.00,8.00) |
| *α_3_* | | Mean: 1.87, 95% CI: 1.72-2.00 | Uniform(0.00,8.00) |
| *α_4_* | | Mean: 1.05, 95% CI: 0.95-1.21 | Uniform(0.00,8.00) |
| *α_5_* | | Mean: 1.64, 95% CI: 1.37-1.77 | Uniform(0.00,8.00) |
| *α_6_* | | Mean: 1.21, 95% CI: 0.76-1.46 | Uniform(0.00,8.00) |
| *α_7_* | | Mean: 2.48, 95% CI: 2.36-2.58 | Uniform(0.00,8.00) |
| Mean number of new infections per day by symptomatic and undiagnosed infected individuals per susceptible population fraction: *K_U0_, K_U1_,…, K_U7_* , where *K_Ui_= σ _i_K_U0_*_,_ for *i=1,…,7.* | | | |
| *K_U0_* | | Mean: 0.21, 95% CI: 0.18-0.25 | Uniform(0.00,5.00) |
| *σ_1_* | | Mean: 3.46, 95% CI: 3.08-3.90 | Uniform(0.00,8.00) |
| *σ_2_* | | Mean: 1.43, 95% CI: 1.15-1.66 | Uniform(0.00,8.00) |
| *σ_3_* | | Mean: 1.13, 95% CI: 0.96-1.26 | Uniform(0.00,8.00) |
| *σ_4_* | | Mean: 1.60, 95% CI: 1.34-1.88 | Uniform(0.00,8.00) |
| *σ_5_* | | Mean: 2.63, 95% CI: 2.41-2.82 | Uniform(0.00,8.00) |
| *σ_6_* | | Mean: 3.74, 95% CI: 3.63-3.94 | Uniform(0.00,8.00) |
| *σ_7_* | | Mean: 1.11, 95% CI: 0.81-1.42 | Uniform(0.00,8.00) |
| Mean number of new infections per day by symptomatic and diagnosed infected individuals per susceptible population fraction: *K_D0_, K_D1_,…, K_D7_*, where *K_Di_= δ_i_K_Ui_*_,_ for *i=0,…,7.* | | | |
| *δ_0_* | | Mean: 0.70, 95% CI: 0.68-0.74 | Uniform(0.00,1.00) |
| *δ_1_* | | Mean: 0.02, 95% CI: 0.00-0.05 | Uniform(0.00,1.00) |
| *δ_2_* | | Mean: 0.72, 95% CI: 0.70-0.75 | Uniform(0.00,1.00) |
| *δ_3_* | | Mean: 0.47, 95% CI: 0.45-0.49 | Uniform(0.00,1.00) |
| *δ_4_* | | Mean: 0.38, 95% CI: 0.37-0.40 | Uniform(0.00,1.00) |
| *δ_5_* | | Mean: 0.44, 95% CI: 0.41-0.47 | Uniform(0.00,1.00) |
| *δ_6_* | | Mean: 0.06, 95% CI: 0.02-0.08 | Uniform(0.00,1.00) |
| *δ_7_* | | Mean: 0.21, 95% CI: 0.16-0.23 | Uniform(0.00,1.00) |
| Probability of recovery of an asymptomatic infected individual, *p_a_*.  Age < 30: *p_a_=* $\bar{p}_{a}$*.*  Age 30-69: *p_a_=* $\gamma_{1}\bar{p}_{a}$*.*  Age 70+: *p_a_=* ${{\gamma_{2}\gamma}_{1}\bar{p}}_{a}$*.* | | | |
| $\bar{p}_{a}$ | | Mean: 0.51, 95% CI: 0.50-0.52 | Uniform(0.50,0.88) |
| $\gamma_{1}$ | | Mean: 0.79, 95% CI: 0.79-0.80 | Uniform(0.50,0.80) |
| $\gamma_{2}$ | | Mean: 0.79, 95% CI: 0.78-0.80 | Uniform(0.50,0.80) |
| Daily probability of recovery of a symptomatic, non-hospitalized individual, *r.*  Age < 30: *r=* $\bar{r}$*.*  Age 30-69: *r=* $\rho_{1}\bar{r}$*.*  Age 70+: *r=* ${\rho_{2}\rho}_{1}\bar{r}$*.* | | | |
| $\bar{r}$ | | Mean: 0.33, 95% CI: 0.30-0.34 | Uniform(0.00,1.00) |
| $\rho_{1}$ | | Mean: 0.98, 95% CI: 0.96-1.00 | Uniform(0.00,1.00) |
| $\rho_{2}$ | | Mean: 0.97, 95% CI: 0.95-0.98 | Uniform(0.00,1.00) |
| Daily probability of recovery of a hospitalized individual, *c.*  Age < 30: *c=* $\bar{c}$*.*  Age 30-69: *c=* $\phi_{1}\bar{c}$*.*  Age 70+: *c=* ${\phi_{2}\phi}_{1}\bar{c}$*.* | | | |
| $\bar{c}$ | | Mean: 0.62, 95% CI: 0.60-0.66 | Uniform(0.00,1.00) |
| $\phi_{1}$ | | Mean: 0.61, 95% CI: 0.57-0.64 | Uniform(0.00,1.00) |
| $\phi_{2}$ | | Mean: 0.23, 95% CI: 0.21-0.27 | Uniform(0.00,1.00) |
| Daily probability of an asymptomatic infected individual developing symptoms, *q.*  Age < 30: *q=*${\psi_{2}\psi}_{1}\bar{q}$*.*  Age 30-69: *q=* $\psi_{1}\bar{q}$*.*  Age 70+: *q=* $\bar{q}$*.* | | | |
| $\bar{q}$ | | Mean: 0.69, 95% CI: 0.67-0.72 | Uniform(0.00,1.00) |
| $\psi_{1}$ | | Mean: 0.81, 95% CI: 0.79-0.83 | Uniform(0.00,1.00) |
| $\psi_{2}$ | | Mean: 0.87, 95% CI: 0.84-0.91 | Uniform(0.00,1.00) |
| Daily probability of diagnosis of an undiagnosed symptomatic individual: *d_0_* and *d_1_*, with *d*_0_= *εd_1_* | | | |
| *d_1_* | | Mean: 0.45, 95% CI: 0.43-0.47 | Uniform(0.00,0.50) |
| *ε* | | Mean: 0.18, 95% CI: 0.17-0.20 | Uniform(0.00,1.00) |
| Daily probability of hospitalization of a diagnosed symptomatic individual, *h* | | | |
| Age <30 | | Mean: 0.03, 95% CI: 0.03-0.03 | Uniform(0.00,0.50) |
| Age 30-69 | | Mean: 0.04, 95% CI: 0.00-0.16 | Uniform(0.00,0.50) |
| Age 70+ | | Mean: 0.26, 95% CI: 0.06-0.44 | Uniform(0.00,0.50) |
| Daily probability of death of a diagnosed, hospitalized individual, *m_h0_ …m_h6_, with* $m_{hj}=m_{h0}\prod_{i=1}^{j} \eta_{i}$*.* | | | |
| *m_h0_* | Age <30 | Mean: 0.00, 95% CI: 0.00-0.00 | Uniform(0.00,0.00) |
|  | Age 30-69 | Mean: 0.22, 95% CI: 0.01-0.43 | Uniform(0.00,0.50) |
|  | Age 70+ | Mean: 0.26, 95% CI: 0.10-0.47 | Uniform(0.00,0.50) |
| *η_1_* | Age <30 | Mean: 0.70, 95% CI: 0.70-0.70 | Uniform(0.40,1.00) |
|  | Age 30-69 | Mean: 0.61, 95% CI: 0.41-0.95 | Uniform(0.40,1.00) |
|  | Age 70+ | Mean: 0.65, 95% CI: 0.43-0.95 | Uniform(0.40,1.00) |
| *η_2_* | Age <30 | Mean: 0.70, 95% CI: 0.70-0.70 | Uniform(0.40,1.00) |
|  | Age 30-69 | Mean: 0.75, 95% CI: 0.50-0.95 | Uniform(0.40,1.00) |
|  | Age 70+ | Mean: 0.62, 95% CI: 0.43-0.90 | Uniform(0.40,1.00) |
| *η_3_* | Age <30 | Mean: 0.70, 95% CI: 0.70-0.70 | Uniform(0.40,1.00) |
|  | Age 30-69 | Mean: 0.73, 95% CI: 0.45-0.97 | Uniform(0.40,1.00) |
|  | Age 70+ | Mean: 0.65, 95% CI: 0.42-0.94 | Uniform(0.40,1.00) |
| *η_4_* | Age <30 | Mean: 0.70, 95% CI: 0.70-0.70 | Uniform(0.40,1.00) |
|  | Age 30-69 | Mean: 0.72, 95% CI: 0.42-0.94 | Uniform(0.40,1.00) |
|  | Age 70+ | Mean: 0.60, 95% CI: 0.41-0.87 | Uniform(0.40,1.00) |
| *η_5_* | Age <30 | Mean: 0.70, 95% CI: 0.70-0.70 | Uniform(0.40,1.00) |
|  | Age 30-69 | Mean: 0.69, 95% CI: 0.44-0.96 | Uniform(0.40,1.00) |
|  | Age 70+ | Mean: 0.67, 95% CI: 0.43-0.96 | Uniform(0.40,1.00) |
| *η_6_* | *Age <30* | Mean: 0.70, 95% CI: 0.70-0.70 | Uniform(0.40,1.00) |
|  | *Age 30-69* | Mean: 0.73, 95% CI: 0.44-0.98 | Uniform(0.40,1.00) |
|  | *Age 70+* | Mean: 0.64, 95% CI: 0.41-0.96 | Uniform(0.40,1.00) |

| **Alberta** | | | |
| --- | --- | --- | --- |
| *Parameter* | | *Posterior* | *Prior* |
| Mean number of new infections per day by symptomatic and undiagnosed infected individuals per susceptible population fraction: *K_A0_, K_A1_,…, K_A7_*, where *K_Ai_= α _i_K_A0_*_,_ for *i=1,…,7.* | | | |
| *K_A0_* | | Mean: 0.05, 95% CI: 0.01-0.09 | Uniform(0.00,5.00) |
| *α_1_* | | Mean: 1.53, 95% CI: 1.30-1.72 | Uniform(0.00,8.00) |
| *α_2_* | | Mean: 1.18, 95% CI: 0.56-1.55 | Uniform(0.00,8.00) |
| *α_3_* | | Mean: 3.61, 95% CI: 3.30-3.79 | Uniform(0.00,8.00) |
| *α_4_* | | Mean: 4.03, 95% CI: 3.69-4.52 | Uniform(0.00,8.00) |
| *α_5_* | | Mean: 2.85, 95% CI: 2.30-3.56 | Uniform(0.00,8.00) |
| *α_6_* | | Mean: 4.81, 95% CI: 4.57-5.07 | Uniform(0.00,8.00) |
| *α_7_* | | Mean: 3.35, 95% CI: 3.09-3.61 | Uniform(0.00,8.00) |
| Mean number of new infections per day by symptomatic and undiagnosed infected individuals per susceptible population fraction: *K_U0_, K_U1_,…, K_U7_* , where *K_Ui_= σ _i_K_U0_*_,_ for *i=1,…,7.* | | | |
| *K_U0_* | | Mean: 0.15, 95% CI: 0.11-0.19 | Uniform(0.00,5.00) |
| *σ_1_* | | Mean: 2.75, 95% CI: 2.35-3.24 | Uniform(0.00,8.00) |
| *σ_2_* | | Mean: 0.28, 95% CI: 0.07-0.50 | Uniform(0.00,8.00) |
| *σ_3_* | | Mean: 1.94, 95% CI: 1.56-2.16 | Uniform(0.00,8.00) |
| *σ_4_* | | Mean: 1.13, 95% CI: 0.91-1.36 | Uniform(0.00,8.00) |
| *σ_5_* | | Mean: 1.13, 95% CI: 0.93-1.31 | Uniform(0.00,8.00) |
| *σ_6_* | | Mean: 1.82, 95% CI: 1.50-2.14 | Uniform(0.00,8.00) |
| *σ_7_* | | Mean: 2.21, 95% CI: 1.93-2.49 | Uniform(0.00,8.00) |
| Mean number of new infections per day by symptomatic and diagnosed infected individuals per susceptible population fraction: *K_D0_, K_D1_,…, K_D7_*, where *K_Di_= δ_i_K_Ui_*_,_ for *i=0,…,7.* | | | |
| *δ_0_* | | Mean: 0.69, 95% CI: 0.61-0.74 | Uniform(0.00,1.00) |
| *δ_1_* | | Mean: 0.17, 95% CI: 0.11-0.20 | Uniform(0.00,1.00) |
| *δ_2_* | | Mean: 0.46, 95% CI: 0.37-0.54 | Uniform(0.00,1.00) |
| *δ_3_* | | Mean: 0.45, 95% CI: 0.41-0.49 | Uniform(0.00,1.00) |
| *δ_4_* | | Mean: 0.41, 95% CI: 0.37-0.44 | Uniform(0.00,1.00) |
| *δ_5_* | | Mean: 0.53, 95% CI: 0.49-0.57 | Uniform(0.00,1.00) |
| *δ_6_* | | Mean: 0.32, 95% CI: 0.28-0.36 | Uniform(0.00,1.00) |
| *δ_7_* | | Mean: 0.27, 95% CI: 0.23-0.31 | Uniform(0.00,1.00) |
| Probability of recovery of an asymptomatic infected individual, *p_a_*.  Age < 30: *p_a_=* $\bar{p}_{a}$*.*  Age 30-69: *p_a_=* $\gamma_{1}\bar{p}_{a}$*.*  Age 70+: *p_a_=* ${{\gamma_{2}\gamma}_{1}\bar{p}}_{a}$*.* | | | |
| $\bar{p}_{a}$ | | Mean: 0.51, 95% CI: 0.50-0.52 | Uniform(0.50,0.88) |
| $\gamma_{1}$ | | Mean: 0.79, 95% CI: 0.79-0.80 | Uniform(0.50,0.80) |
| $\gamma_{2}$ | | Mean: 0.79, 95% CI: 0.78-0.80 | Uniform(0.50,0.80) |
| Daily probability of recovery of a symptomatic, non-hospitalized individual, *r.*  Age < 30: *r=* $\bar{r}$*.*  Age 30-69: *r=* $\rho_{1}\bar{r}$*.*  Age 70+: *r=* ${\rho_{2}\rho}_{1}\bar{r}$*.* | | | |
| $\bar{r}$ | | Mean: 0.08, 95% CI: 0.05-0.10 | Uniform(0.00,1.00) |
| $\rho_{1}$ | | Mean: 0.96, 95% CI: 0.92-0.99 | Uniform(0.00,1.00) |
| $\rho_{2}$ | | Mean: 0.97, 95% CI: 0.91-0.99 | Uniform(0.00,1.00) |
| Daily probability of recovery of a hospitalized individual, *c.*  Age < 30: *c=* $\bar{c}$*.*  Age 30-69: *c=* $\phi_{1}\bar{c}$*.*  Age 70+: *c=* ${\phi_{2}\phi}_{1}\bar{c}$*.* | | | |
| $\bar{c}$ | | Mean: 0.83, 95% CI: 0.74-0.88 | Uniform(0.00,1.00) |
| $\phi_{1}$ | | Mean: 0.69, 95% CI: 0.65-0.73 | Uniform(0.00,1.00) |
| $\phi_{2}$ | | Mean: 0.46, 95% CI: 0.44-0.50 | Uniform(0.00,1.00) |
| Daily probability of an asymptomatic infected individual developing symptoms, *q.*  Age < 30: *q=*${\psi_{2}\psi}_{1}\bar{q}$*.*  Age 30-69: *q=* $\psi_{1}\bar{q}$*.*  Age 70+: *q=* $\bar{q}$*.* | | | |
| $\bar{q}$ | | Mean: 0.63, 95% CI: 0.61-0.66 | Uniform(0.00,1.00) |
| $\psi_{1}$ | | Mean: 0.89, 95% CI: 0.87-0.94 | Uniform(0.00,1.00) |
| $\psi_{2}$ | | Mean: 0.85, 95% CI: 0.82-0.89 | Uniform(0.00,1.00) |
| Daily probability of diagnosis of an undiagnosed symptomatic individual: *d_0_* and *d_1_*, with *d*_0_= *εd_1_* | | | |
| *d_1_* | | Mean: 0.23, 95% CI: 0.21-0.26 | Uniform(0.00,0.50) |
| *ε* | | Mean: 0.58, 95% CI: 0.53-0.64 | Uniform(0.00,1.00) |
| Daily probability of hospitalization of a diagnosed symptomatic individual, *h* | | | |
| Age <30 | | Mean: 0.63, 95% CI: 0.56-0.69 | Uniform(0.00,1.00) |
| Age 30-69 | | Mean: 0.90, 95% CI: 0.82-0.94 | Uniform(0.00,1.00) |
| Age 70+ | | Mean: 0.88, 95% CI: 0.85-0.92 | Uniform(0.00,1.00) |
| Daily probability of death of a diagnosed, hospitalized individual, *m_h0_ …m_h6_, with* $m_{hj}=m_{h0}\prod_{i=1}^{j} \eta_{i}$*.* | | | |
| *m_h0_* | Age <30 | Mean: 0.00, 95% CI: 0.00-0.00 | Uniform(0.00,0.00) |
|  | Age 30-69 | Mean: 0.06, 95% CI: 0.00-0.14 | Uniform(0.00,0.20) |
|  | Age 70+ | Mean: 0.11, 95% CI: 0.02-0.19 | Uniform(0.00,0.20) |
| *η_1_* | Age <30 | Mean: 0.70, 95% CI: 0.70-0.70 | Uniform(0.40,1.00) |
|  | Age 30-69 | Mean: 0.67, 95% CI: 0.42-0.94 | Uniform(0.40,1.00) |
|  | Age 70+ | Mean: 0.76, 95% CI: 0.48-0.98 | Uniform(0.40,1.00) |
| *η_2_* | Age <30 | Mean: 0.70, 95% CI: 0.70-0.70 | Uniform(0.40,1.00) |
|  | Age 30-69 | Mean: 0.70, 95% CI: 0.45-0.94 | Uniform(0.40,1.00) |
|  | Age 70+ | Mean: 0.69, 95% CI: 0.44-0.97 | Uniform(0.40,1.00) |
| *η_3_* | Age <30 | Mean: 0.70, 95% CI: 0.70-0.70 | Uniform(0.40,1.00) |
|  | Age 30-69 | Mean: 0.72, 95% CI: 0.44-0.98 | Uniform(0.40,1.00) |
|  | Age 70+ | Mean: 0.70, 95% CI: 0.43-0.97 | Uniform(0.40,1.00) |
| *η_4_* | Age <30 | Mean: 0.70, 95% CI: 0.70-0.70 | Uniform(0.40,1.00) |
|  | Age 30-69 | Mean: 0.67, 95% CI: 0.42-0.95 | Uniform(0.40,1.00) |
|  | Age 70+ | Mean: 0.71, 95% CI: 0.48-0.95 | Uniform(0.40,1.00) |
| *η_5_* | Age <30 | Mean: 0.70, 95% CI: 0.70-0.70 | Uniform(0.40,1.00) |
|  | Age 30-69 | Mean: 0.63, 95% CI: 0.42-0.92 | Uniform(0.40,1.00) |
|  | Age 70+ | Mean: 0.75, 95% CI: 0.49-0.97 | Uniform(0.40,1.00) |
| *η_6_* | *Age <30* | Mean: 0.70, 95% CI: 0.70-0.70 | Uniform(0.40,1.00) |
|  | *Age 30-69* | Mean: 0.65, 95% CI: 0.42-0.95 | Uniform(0.40,1.00) |
|  | *Age 70+* | Mean: 0.68, 95% CI: 0.42-0.98 | Uniform(0.40,1.00) |

| **British Columbia** | | | |
| --- | --- | --- | --- |
| *Parameter* | | *Posterior* | *Prior* |
| Mean number of new infections per day by symptomatic and undiagnosed infected individuals per susceptible population fraction: *K_A0_, K_A1_,…, K_A7_*, where *K_Ai_= α _i_K_A0_*_,_ for *i=1,…,7.* | | | |
| *K_A0_* | | Mean: 0.67, 95% CI: 0.64-0.69 | Uniform(0.00,5.00) |
| *α_1_* | | Mean: 0.19, 95% CI: 0.17-0.22 | Uniform(0.00,8.00) |
| *α_2_* | | Mean: 0.25, 95% CI: 0.21-0.29 | Uniform(0.00,8.00) |
| *α_3_* | | Mean: 0.52, 95% CI: 0.47-0.61 | Uniform(0.00,8.00) |
| *α_4_* | | Mean: 0.69, 95% CI: 0.63-0.77 | Uniform(0.00,8.00) |
| *α_5_* | | Mean: 0.33, 95% CI: 0.30-0.38 | Uniform(0.00,8.00) |
| *α_6_* | | Mean: 0.89, 95% CI: 0.85-0.91 | Uniform(0.00,8.00) |
| *α_7_* | | Mean: 0.61, 95% CI: 0.58-0.63 | Uniform(0.00,8.00) |
| Mean number of new infections per day by symptomatic and undiagnosed infected individuals per susceptible population fraction: *K_U0_, K_U1_,…, K_U7_* , where *K_Ui_= σ _i_K_U0_*_,_ for *i=1,…,7.* | | | |
| *K_U0_* | | Mean: 0.03, 95% CI: 0.03-0.04 | Uniform(0.00,5.00) |
| *σ_1_* | | Mean: 1.02, 95% CI: 0.99-1.04 | Uniform(0.00,8.00) |
| *σ _2_* | | Mean: 1.76, 95% CI: 1.73-1.79 | Uniform(0.00,8.00) |
| *σ_3_* | | Mean: 2.71, 95% CI: 2.66-2.75 | Uniform(0.00,8.00) |
| *σ_4_* | | Mean: 2.39, 95% CI: 2.33-2.42 | Uniform(0.00,8.00) |
| *σ_5_* | | Mean: 3.25, 95% CI: 3.19-3.37 | Uniform(0.00,8.00) |
| *σ_6_* | | Mean: 0.57, 95% CI: 0.52-0.63 | Uniform(0.00,8.00) |
| *σ_7_* | | Mean: 2.97, 95% CI: 2.93-3.01 | Uniform(0.00,8.00) |
| Mean number of new infections per day by symptomatic and diagnosed infected individuals per susceptible population fraction: *K_D0_, K_D1_,…, K_D7_*, where *K_Di_= δ_i_K_Ui_*_,_ for *i=0,…,7.* | | | |
| *δ_0_* | | Mean: 0.48, 95% CI: 0.46-0.49 | Uniform(0.00,1.00) |
| *δ_1_* | | Mean: 0.54, 95% CI: 0.54-0.55 | Uniform(0.00,1.00) |
| *δ_2_* | | Mean: 0.41, 95% CI: 0.40-0.42 | Uniform(0.00,1.00) |
| *δ_3_* | | Mean: 0.48, 95% CI: 0.47-0.49 | Uniform(0.00,1.00) |
| *δ_4_* | | Mean: 0.54, 95% CI: 0.53-0.54 | Uniform(0.00,1.00) |
| *δ_5_* | | Mean: 0.44, 95% CI: 0.44-0.45 | Uniform(0.00,1.00) |
| *δ_6_* | | Mean: 0.57, 95% CI: 0.56-0.57 | Uniform(0.00,1.00) |
| *δ_7_* | | Mean: 0.39, 95% CI: 0.39-0.40 | Uniform(0.00,1.00) |
| Probability of recovery of an asymptomatic infected individual, *p_a_*.  Age < 30: *p_a_=* $\bar{p}_{a}$*.*  Age 30-69: *p_a_=* $\gamma_{1}\bar{p}_{a}$*.*  Age 70+: *p_a_=* ${{\gamma_{2}\gamma}_{1}\bar{p}}_{a}$*.* | | | |
| $\bar{p}_{a}$ | | Mean: 0.50, 95% CI: 0.50-0.50 | Uniform(0.50,0.88) |
| $\gamma_{1}$ | | Mean: 0.79, 95% CI: 0.79-0.80 | Uniform(0.50,0.80) |
| $\gamma_{2}$ | | Mean: 0.80, 95% CI: 0.79-0.80 | Uniform(0.50,0.80) |
| Daily probability of recovery of a symptomatic, non-hospitalized individual, *r.*  Age < 30: *r=* $\bar{r}$*.*  Age 30-69: *r=* $\rho_{1}\bar{r}$*.*  Age 70+: *r=* ${\rho_{2}\rho}_{1}\bar{r}$*.* | | | |
| $\bar{r}$ | | Mean: 0.08, 95% CI: 0.07-0.09 | Uniform(0.00,1.00) |
| $\rho_{1}$ | | Mean: 0.99, 95% CI: 0.99-1.00 | Uniform(0.00,1.00) |
| $\rho_{2}$ | | Mean: 0.99, 95% CI: 0.99-1.00 | Uniform(0.00,1.00) |
| Daily probability of recovery of a hospitalized individual, *c.*  Age < 30: *c=* $\bar{c}$*.*  Age 30-69: *c=* $\phi_{1}\bar{c}$*.*  Age 70+: *c=* ${\phi_{2}\phi}_{1}\bar{c}$*.* | | | |
| $\bar{c}$ | | Mean: 0.52, 95% CI: 0.51-0.52 | Uniform(0.00,1.00) |
| $\phi_{1}$ | | Mean: 0.58, 95% CI: 0.57-0.58 | Uniform(0.00,1.00) |
| $\phi_{2}$ | | Mean: 0.40, 95% CI: 0.40-0.41 | Uniform(0.00,1.00) |
| Daily probability of an asymptomatic infected individual developing symptoms, *q.*  Age < 30: *q=*${\psi_{2}\psi}_{1}\bar{q}$*.*  Age 30-69: *q=* $\psi_{1}\bar{q}$*.*  Age 70+: *q=* $\bar{q}$*.* | | | |
| $\bar{q}$ | | Mean: 0.41, 95% CI: 0.40-0.41 | Uniform(0.00,1.00) |
| $\psi_{1}$ | | Mean: 0.90, 95% CI: 0.89-0.91 | Uniform(0.00,1.00) |
| $\psi_{2}$ | | Mean: 0.87, 95% CI: 0.87-0.89 | Uniform(0.00,1.00) |
| Daily probability of diagnosis of an undiagnosed symptomatic individual: *d_0_* and *d_1_*, with *d*_0_= *εd_1_* | | | |
| *d_1_* | | Mean: 0.08, 95% CI: 0.08-0.09 | Uniform(0.00,0.50) |
| *ε* | | Mean: 0.00, 95% CI: 0.00-0.01 | Uniform(0.00,1.00) |
| Daily probability of hospitalization of a diagnosed symptomatic individual, *h* | | | |
| Age <30 | | Mean: 0.01, 95% CI: 0.01-0.01 | Uniform(0.00,0.05) |
| Age 30-69 | | Mean: 0.01, 95% CI: 0.00-0.04 | Uniform(0.00,0.05) |
| Age 70+ | | Mean: 0.03, 95% CI: 0.01-0.05 | Uniform(0.00,0.05) |
| Daily probability of death of a diagnosed, hospitalized individual, *m_h0_ …m_h6_, with* $m_{hj}=m_{h0}\prod_{i=1}^{j} \eta_{i}.$ | | | |
| *m_h0_* | Age <30 | Mean: 0.00, 95% CI: 0.00-0.00 | Uniform(0.00,0.20) |
|  | Age 30-69 | Mean: 0.08, 95% CI: 0.01-0.19 | Uniform(0.00,0.20) |
|  | Age 70+ | Mean: 0.12, 95% CI: 0.04-0.19 | Uniform(0.00,0.20) |
| *η_1_* | Age <30 | Mean: 0.70, 95% CI: 0.70-0.70 | Uniform(0.40,1.00) |
|  | Age 30-69 | Mean: 0.68, 95% CI: 0.41-0.97 | Uniform(0.40,1.00) |
|  | Age 70+ | Mean: 0.70, 95% CI: 0.41-0.99 | Uniform(0.40,1.00) |
| *η_2_* | Age <30 | Mean: 0.70, 95% CI: 0.70-0.70 | Uniform(0.40,1.00) |
|  | Age 30-69 | Mean: 0.68, 95% CI: 0.44-0.97 | Uniform(0.40,1.00) |
|  | Age 70+ | Mean: 0.70, 95% CI: 0.42-0.98 | Uniform(0.40,1.00) |
| *η_3_* | Age <30 | Mean: 0.70, 95% CI: 0.70-0.70 | Uniform(0.40,1.00) |
|  | Age 30-69 | Mean: 0.71, 95% CI: 0.43-0.98 | Uniform(0.40,1.00) |
|  | Age 70+ | Mean: 0.66, 95% CI: 0.41-0.98 | Uniform(0.40,1.00) |
| *η_4_* | Age <30 | Mean: 0.70, 95% CI: 0.70-0.70 | Uniform(0.40,1.00) |
|  | Age 30-69 | Mean: 0.67, 95% CI: 0.42-0.98 | Uniform(0.40,1.00) |
|  | Age 70+ | Mean: 0.67, 95% CI: 0.41-0.96 | Uniform(0.40,1.00) |
| *η_5_* | Age <30 | Mean: 0.70, 95% CI: 0.70-0.70 | Uniform(0.40,1.00) |
|  | Age 30-69 | Mean: 0.71, 95% CI: 0.45-0.95 | Uniform(0.40,1.00) |
|  | Age 70+ | Mean: 0.69, 95% CI: 0.42-0.99 | Uniform(0.40,1.00) |
| *η_6_* | *Age <30* | Mean: 0.70, 95% CI: 0.70-0.70 | Uniform(0.40,1.00) |
|  | *Age 30-69* | Mean: 0.70, 95% CI: 0.42-0.97 | Uniform(0.40,1.00) |
|  | *Age 70+* | Mean: 0.69, 95% CI: 0.43-0.99 | Uniform(0.40,1.00) |
