# Supplementary material for: Estimation of COVID-19 Period Prevalence and the Undiagnosed Population in Canadian Provinces: Model-Based Analysis
Source: JMIR Public Health Surveill. 2021 Sep 9;7(9):e26409. doi: 10.2196/26409 (PMC8432517; doi:10.2196/26409)
Supplement: Multimedia Appendix 3 [file publichealth_v7i9e26409_app3.docx]

**Appendix 3:** Model Validation Results - Estimated trajectories and reported numbers of COVID-19 diagnoses and deaths between March 1 and July 21, 2020. Red dots represent daily observed cases (i.e. diagnoses and deaths). Each green line represents the simulated mean trajectory of the model conditional on model parameters sampled by the MCMC algorithm. Dotted blue lines represent the 95% credible band (CB) of the mean trajectories. Solid blue lines represent the median of all simulated mean trajectories. As there was only one reported COVID-19 death among the under-30 age group (in Alberta), we do not estimate the mean number of deaths for this cohort.

**Diagnoses**

| **Quebec** | **Ontario** | **Alberta** | **British Columbia** |
| --- | --- | --- | --- |
| **Age <30** |  |  |  |
| 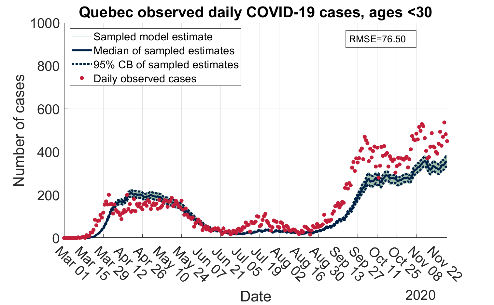 | 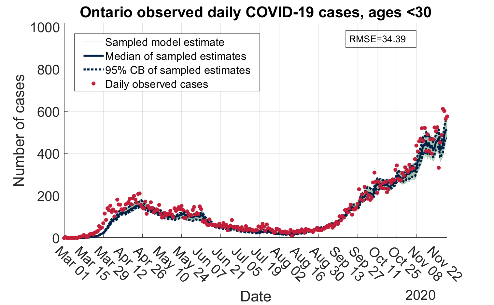 | 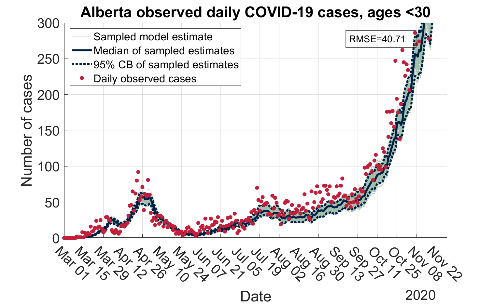 | 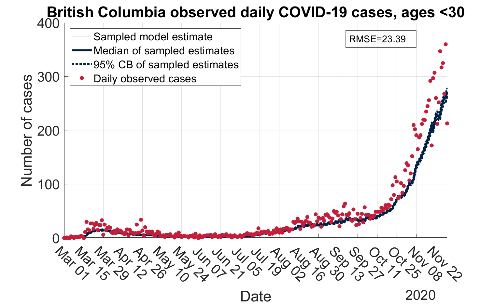 |
| **Age 30-69** |  |  |  |
| 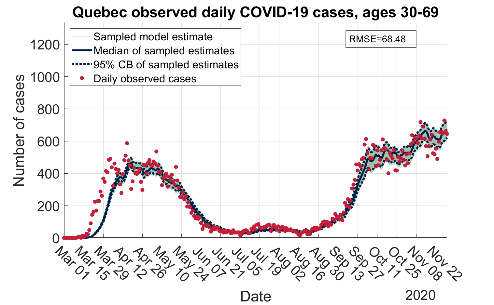 | 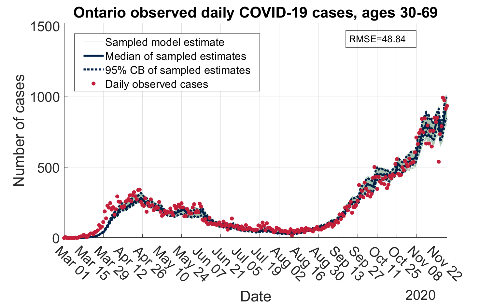 | 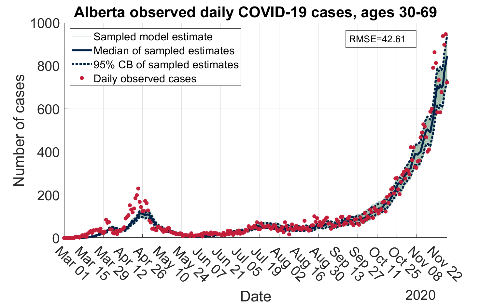 | 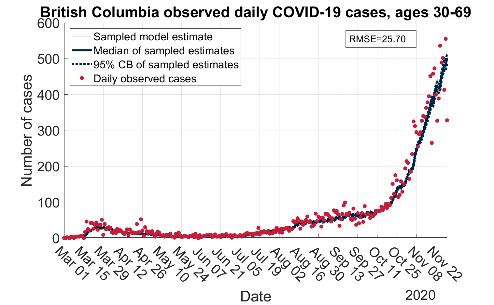 |
| **Age 70+** |  |  |  |
| 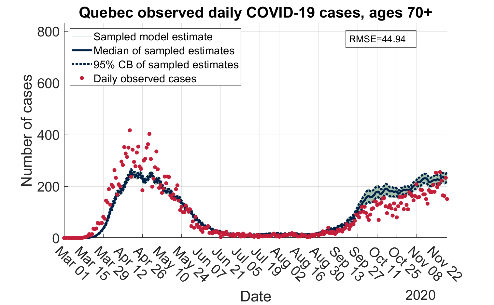 | 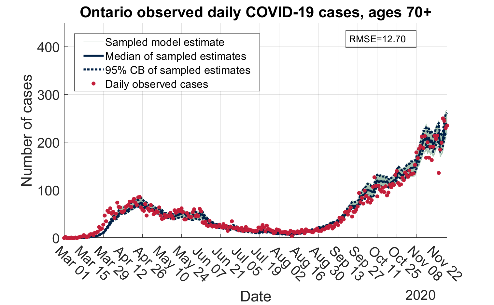 | 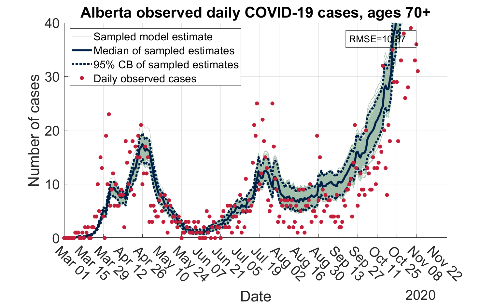 | 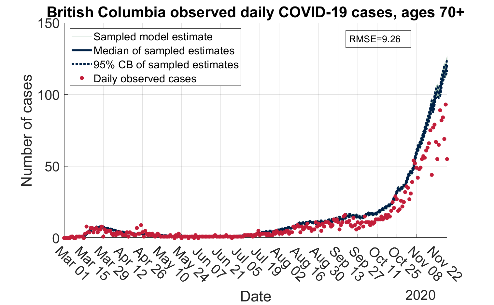 |

**Deaths**

| Quebec | Ontario | Alberta | British Columbia |
| --- | --- | --- | --- |
| Age 30-69 |  |  |  |
| 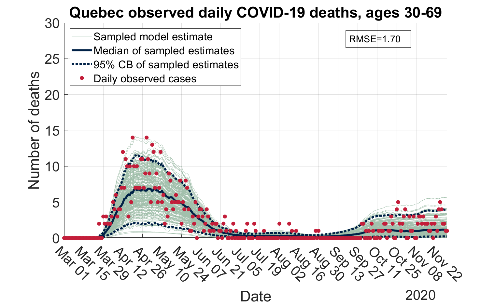 | 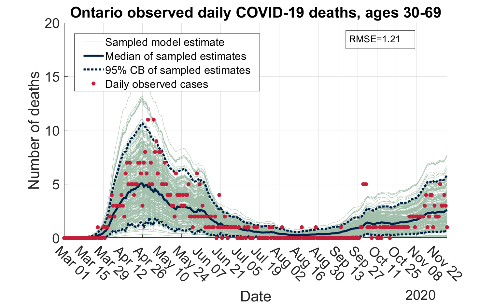 | 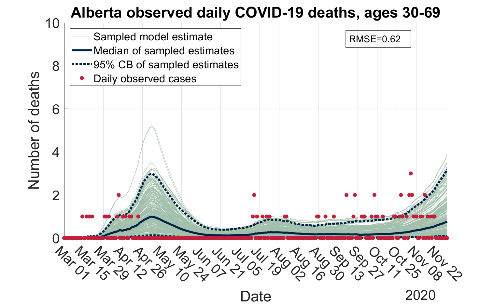 | 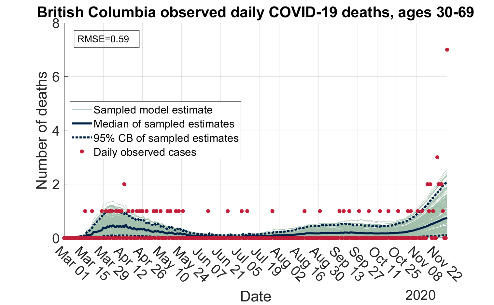 |
| Age 70+ |  |  |  |
| 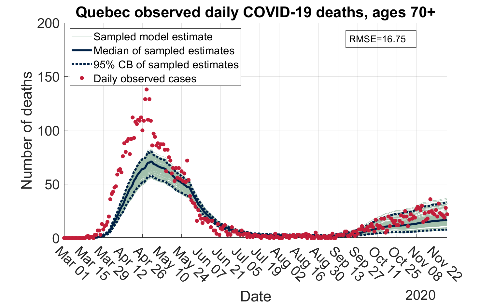 | 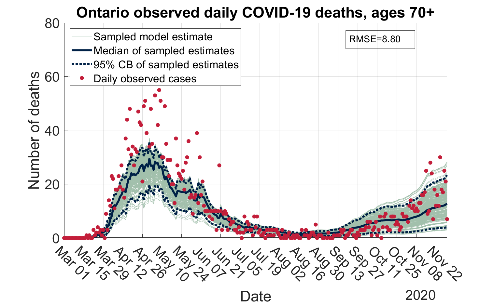 | 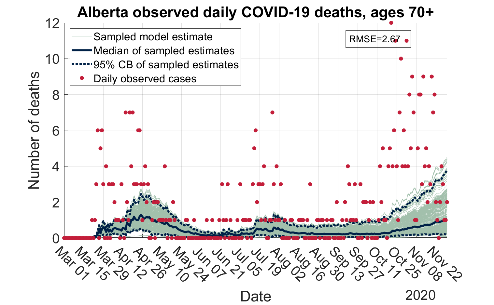 | 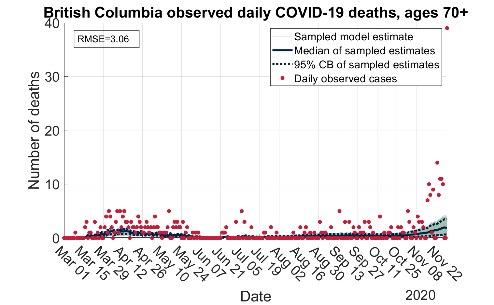 |
