# Supplementary material for: Estimation of COVID-19 Period Prevalence and the Undiagnosed Population in Canadian Provinces: Model-Based Analysis
Source: JMIR Public Health Surveill. 2021 Sep 9;7(9):e26409. doi: 10.2196/26409 (PMC8432517; doi:10.2196/26409)
Supplement: Multimedia Appendix 4 [file publichealth_v7i9e26409_app4.docx]

**Appendix 4:** Distribution of the estimated cumulative total number of COVID-19 cases, reported cumulative diagnoses and reported cumulative deaths.

| **Quebec** | **Ontario** | **Alberta** | **British Columbia** |
| --- | --- | --- | --- |
| **Age <30** |  |  |  |
| 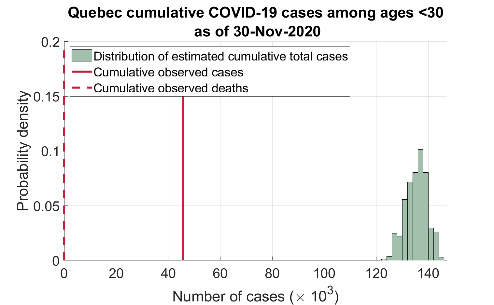 | 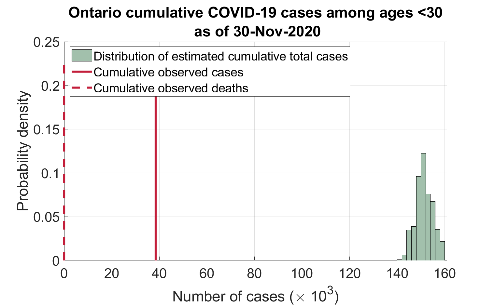 | 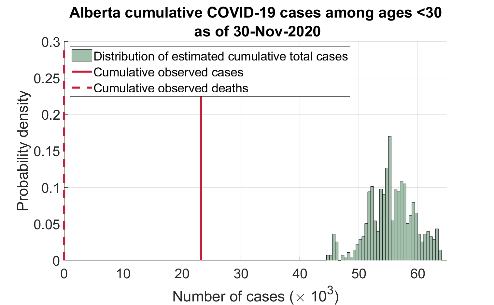 | 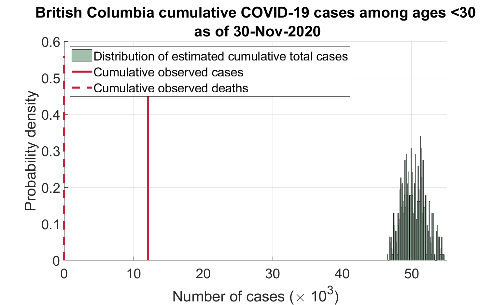 |
| **Age 30-69** |  |  |  |
| 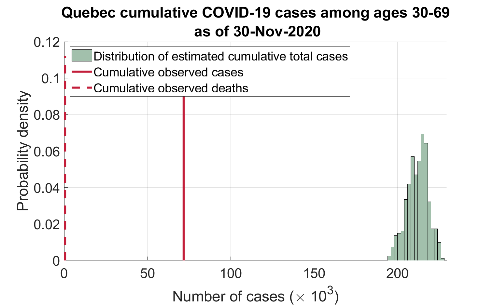 | 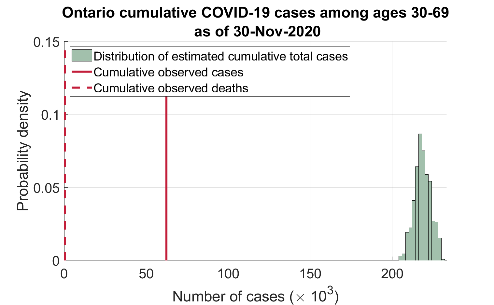 | 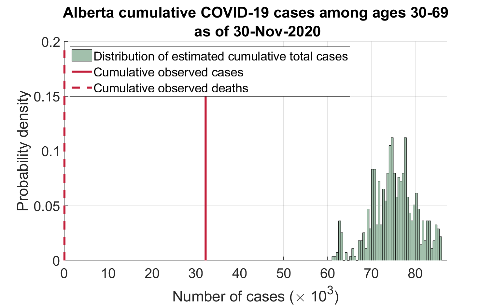 | 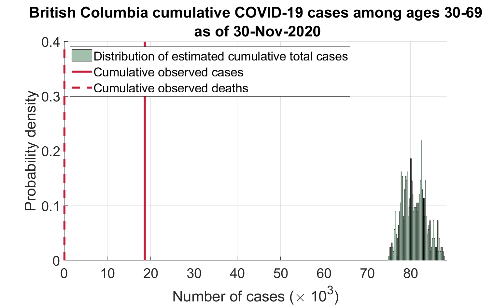 |
| **Age 70+** |  |  |  |
| 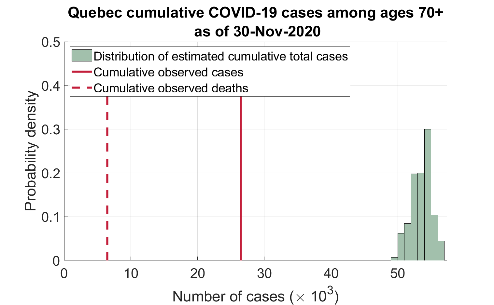 | 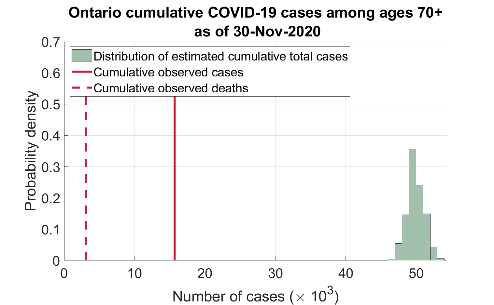 | 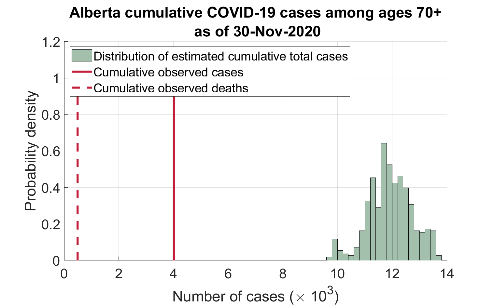 | 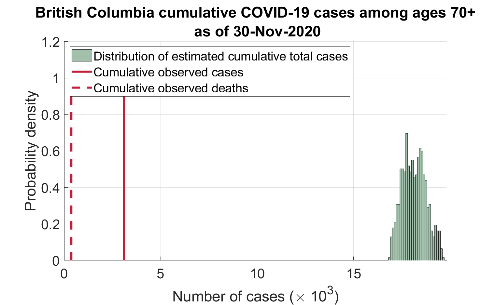 |
