# Supplementary material for: Estimation of COVID-19 Period Prevalence and the Undiagnosed Population in Canadian Provinces: Model-Based Analysis
Source: JMIR Public Health Surveill. 2021 Sep 9;7(9):e26409. doi: 10.2196/26409 (PMC8432517; doi:10.2196/26409)
Supplement: Multimedia Appendix 5 [file publichealth_v7i9e26409_app5.docx]

**Appendix 5:** Estimated cumulative total COVID-19 infections on November 30, 2020.

| **Province** | **Age cohort** | **Estimated total infections between March 1 and November 30, 2020** | | |
| --- | --- | --- | --- | --- |
|  |  | **Median** | **Mean** | **95% CI** |
| **Quebec** | Age <30 | 135780 | 135407 | 126380-143185 |
|  | Age 30-69 | 212774 | 212048 | 198212-223863 |
|  | Age 70+ | 53753 | 53549 | 50462-56298 |
| **Ontario** | Age <30 | 151221 | 151443 | 144707-158804 |
|  | Age 30-69 | 218268 | 218446 | 208519-228609 |
|  | Age 70+ | 49823 | 49937 | 47614-52440 |
| **Alberta** | Age <30 | 55441 | 55596 | 45892-63063 |
|  | Age 30-69 | 75115 | 75246 | 62932-85136 |
|  | Age 70+ | 11892 | 11932 | 9961-13445 |
| **British Columbia** | Age <30 | 50282 | 50356 | 47318-53912 |
|  | Age 30-69 | 80703 | 80915 | 76063-86605 |
|  | Age 70+ | 18124 | 18147 | 17076-19397 |
